# Improving psychosocial distress for young adolescents in rural schools of Pakistan: study protocol of a cluster randomised controlled trial

**DOI:** 10.1136/bmjopen-2022-063607

**Published:** 2022-09-23

**Authors:** Syed Usman Hamdani, Zill-E- Huma, Aiysha Malik, Asad Tamizuddin- Nizami, Um ul Baneen, Nadia Suleman, Hashim Javed, Duolao Wang, Mark van Ommeren, Samra Mazhar, Shahzad Alam Khan, Fareed Aslam Minhas, Atif Rahman

**Affiliations:** 1 Global Institute of Human Development, Shifa Tameer-e-Millat University, Islamabad, Pakistan; 2 Department of Primary Care and Mental Health, University of Liverpool Faculty of Health and Life Sciences, Liverpool, UK; 3 Child and Adolescent Mental Health, Human Development Research Foundation, Islamabad, Pakistan; 4 Department of Mental Health and Substance Use, World Health Organization, Geneve, Switzerland; 5 Institute of Psychiatry, Benazir Bhutto Hospital, Rawalpindi, Pakistan; 6 Department of Clinical Sciences, Liverpool School of Tropical Medicine, Liverpool, UK; 7 Noncommunicable Diseases and Mental Health Department, Pakistan Ministry of National Health Services Regulations and Coordination, Islamabad, Pakistan; 8 Noncommunicable Diseases and Mental Health Department, World Health Organization, Islamabad, Pakistan

**Keywords:** child & adolescent psychiatry, depression & mood disorders, mental health, anxiety disorders

## Abstract

**Introduction:**

Emotional problems are leading contributors to health burden among adolescents worldwide. There is an urgent need for evidence-based psychological interventions for young people. This study aims to evaluate the effectiveness of a school-based, group psychological intervention, Early Adolescent Skills for Emotions (EASE) developed by the WHO to improve psychosocial distress in Pakistani adolescents.

**Method and analysis:**

A two-arm, single-blinded, cluster randomised controlled trial, with a wait-list control arm is being conducted in school settings of rural Pakistan. Forty eligible public-school clusters have been randomised (stratified by gender) on a 1:1 allocation ratio into intervention (n=20) and control arm (n=20). Following informed consent, 564 adolescents with psychosocial distress (Youth-reported Paediatric Symptoms Checklist, cut-off ≥28) from 40 schools have been enrolled into the trial (14±3 average cluster size) between 2 November 2021 and 30th November 2021. Participants in the intervention arm will receive EASE in 7-weekly adolescents and 3-biweekly caregivers group sessions in schools. The adolescent sessions involve the components of psychoeducation, stress management, behavioural activation, problem-solving and relapse prevention. Caregivers will receive training to learn and implement active listening; spending quality time and using praise as a strategy to help their children. The primary outcome is reduction in psychosocial distress at 3 months postintervention. Secondary outcomes include symptoms of depression and anxiety, caregiver–adolescent relationship and caregivers’ well-being. Outcomes will be assessed at baseline, immediate 1 week and 3-months postintervention. Qualitative process evaluation will explore barriers and facilitators to programme implementation in low-resource school settings.

**Ethics:**

Ethics approval has been obtained from Central Ethics Committee of University of Liverpool, UK, Ethics Review Committee of WHO Geneva and from the Institutional Review Board of Human Development Research Foundation (HDRF), Pakistan.

**Dissemination:**

The findings of the study will be disseminated by WHO and through peer-reviewed publications.

**Trial registration number:**

ISRCTN17755448.

**STRENGTHS AND LIMITATIONS OF THIS STUDY**

- To help young adolescents with internalising problems, a transdiagnostic psychological intervention was developed by WHO called ‘Early Adolescent Skills for Emotions (EASE)’. The intervention is designed to be delivered through non-specialist facilitators in low-resource settings.
- A two-arm, single-blinded, cluster randomised controlled trial, with a wait-list control arm, adequate sample size and power and an embedded qualitative process evaluation is being conducted to evaluate the effectiveness and cost-effectiveness of EASE in school settings of Pakistan.
- The study is being conducted in one rural geographical area of Pakistan and may need more studies in other areas for generalisability.
- The study uses self-reported measures for most outcomes. However, these are considered standard and used widely in the field.

## Introduction

Schools are an important public health platform to promote positive youth mental health globally.^1 2^ Schools are uniquely placed to reach significant numbers of young people to address their mental health needs, especially in low-income and middle-income countries (LMICs), where a lack of child and adolescent mental health services and experts; lack of access to mental health services; low awareness of mental health, and economic and societal barriers such as stigma remain key challenges to the provision of evidence-based mental health services.^3^ There is growing evidence from both high income and LMICs that school-based mental health programmes are associated with beneficial mental health outcomes in adolescents.^1 2^ However, scale-up and sustainable implementation of mental health programmes for young people in LMICs demands political will, stakeholder involvement, intersectoral coordination and leadership often between health and education sectors,^4^ particularly in a post-COVID context, where 91% of the world’s student population has been negatively impacted by closures of schools due to COVID-19 pandemic.^5^

Rates of anxiety and depression in young people have been exacerbated during COVID-19 pandemic.^6^ Moreover, due to exacerbation of psychosocial stressors including alteration in daily routine and social interactions during COVID-19 pandemic, at-risk adolescents are more likely to develop severe psychological problems. This situation demands an urgent need to provide more effective mental health support to school going adolescents to ensure that young people with symptoms of distress have access to the support that they need in their schools.

Youth in Pakistan account for 35% of its population (below the age of 14 years) and are exposed to a number of chronic adversities such as poverty, violence and socio and economic inequalities which makes them more susceptible to develop mental health problems early in their adolescence.^7^ In addition, the ongoing COVID-19 pandemic situation is adversely affecting the economic, social and emotional well-being of the population at large and is particularly negatively impacting the well-being of adolescents due to nationwide lockdowns, closure of schools and disruption of academic year.^8^ A recent epidemiological study, conducted pre-COVID-19, with 5856 adolescents from 41 public schools in rural Pakistan reported 25% prevalence rate of psychosocial distress in school going adolescents.^9^

Recognising the ever-growing burden of adolescent mental health problems in Pakistan, the Ministry of Health in Pakistan has launched the President’s programme to promote youth mental health through schools.^10^ It emphasises the role of early-life interventions to promote mental health and prevent mental illnesses and takes a multitiered approach and recommends training non-specialists such as ‘university graduates’ to promote socioemotional well-being of school-going adolescents. Under the ambit of President’ Programme, Early Adolescents Skills for Emotions (EASE), is being implemented in public schools of rural Pakistan to address internalising problems in school-going adolescents. EASE was developed by WHO as a brief, transdiagnostic group psychological intervention designed to be delivered by non-specialists, to help young people aged approximately 10 to 14 years experiencing internalising problems such as psychosocial distress and symptoms of depression and anxiety^11^ (for more details on intervention please see section on interventions below). EASE has been previously translated in Urdu (National Language of Pakistan), culturally adapted for implementation in public schools and feasibility tested in eight public schools of rural Pakistan using a feasibility cluster randomised controlled trial (cRCT) design (trial registration NCT04254393). The results of the evaluation demonstrated the acceptability and feasibility of the adapted intervention to be delivered by non-specialist facilitators in public school settings of Pakistan and exhibited promising effects on improving adolescent’s well-being.^12^

This study aims to evaluate the effectiveness and cost-effectiveness of the culturally adapted EASE intervention compared with wait-list control to improve psychosocial distress in adolescents and improve caregiver-adolescent relationship and caregivers’ well-being at 3 months postintervention in public school settings of Pakistan.

### Hypotheses

Our primary hypothesis is that *EASE* will be superior compared with *waitlist control*, in reducing the psychosocial distress in adolescents (aged 13–15 years), measured with the self-rated Paediatric Symptom Checklist (PSC) at 3 months’ postintervention. Our secondary hypotheses are that *EASE* will result in improving adolescent well-being, quality of life, problem-solving skills, perceived emotional support, caregiver–adolescents relationship and caregivers’ well-being and reducing somatic complaints and anxiety and depressive symptoms in adolescents.

## Methods and analysis

### Study design

A two-arm, single-blind, cRCT, with a wait-list control arm and an embedded qualitative study is being conducted in public schools of rural subdistrict of Gujar Khan in Rawalpindi, Pakistan. The unit of randomisation is a school. In this study, 40 eligible school clusters, stratified by gender, have been randomised into intervention and control arms with a 1:1 allocation ratio. Outcomes will be assessed at baseline and 1 week and 3 months postintervention.

### Patient and public involvement

The research team has culturally adapted and feasibility tested the intervention by working collaboratively with the adolescents, caregivers and school administration from the same study subdistrict. As a part of the formative phase we conducted (1) qualitative needs assessments with school adolescents to identify the priority adolescent mental health problems; (2) end-user testing workshops with adolescents in school settings to culturally adapt the EASE intervention and (3) consultative workshops with relevant stakeholders from Ministries of education and health of Pakistan, school staff including head teachers, teachers and mental health experts, parents and adolescents to develop a hypothesised pathway for the implementation of school based mental health programmes in low resource settings of Pakistan.^13^ Once the current trial is complete, the findings will be disseminated to participants, ministries of health and education, school education department and wider public through presentations at community and public forums.

### Study settings

The study will be conducted in 40 middle and high public schools of rural subdistrict of Gujar Khan, located in the Rawalpindi district in the province of Punjab in Pakistan (approx. population of 1000 000). It is a pilot site for the implementation of President’s Mental Health Programme and falls under the catchment area of Institute of Psychiatry (IoP), Benazir Bhutto Hospital, Rawalpindi which is the sponsoring institute of this study. The subdistrict is semirural, with agrarian-based economy and represents typical rural area in the country. The population speaks Punjabi with Potohari being the predominant dialect. In Gujar Khan subdistrict, there are 497 public schools (323-primary, 89 middle and 85 high schools). There are 231 schools for boys and 266 schools for girls in Gujar Khan in total. The primary decision body for public schools in Gujar Khan is District Education Department and with its permission 40 public schools (20 boys and 20 girls schools) were included in this study. Literacy rates in the study district are 80%.^14^

Mental health services in Pakistan are provided through specialist mental health units at tertiary healthcare facilities, concentred in urban centres with little or no mental healthcare for rural populations. School health services in public schools are provided through School Health and Nutrition Supervisors, who are based at primary healthcare (PHC) centres and visit schools once a month to screen students for Eye, ears, nose and throat, dental, skin and general physical problems and if any health problems are identified, the students are referred to the medical officer of the concerned PHC centres.

### Research participants

The age range of adolescents in school studying in academic grades 8–10 are 13–15 years of age. Our formative work indicated that challenges faced by adolescents in grades 8–10 include academic stress, expectations of high academic achievements from parents and teachers, peer pressure, interpersonal problems, worries about the future and stressful home environment (publication forthcoming). These stressors often lead to mental health problems including distress, anxiety and depression like symptoms among adolescents. The need for focused psychological support for the mental health of adolescents in this age group has been identified as a priority by the education sector stakeholders.

The research participants for the current study are adolescents, screened positive for psychosocial distress with cut-off score of ≥28 on self-rated PSC (validated cut-off score for school going adolescents in Pakistan).^9^

### Eligibility criteria of participants

#### Inclusion criteria

- Adolescents aged 13–15 years.
- Living with parents/primary caregivers, attending public middle and high schools in the Gujar Khan sub-district of Rawalpindi, Pakistan.
- Written parent/primary caregiver informed consent or witnessed consent and adolescent assent for participation in the study.
- Screened positive on self-reported PSC (cut- off score ≥28).
- Where there is more than one eligible child in a family unit, we will include the youngest eligible child.

#### Exclusion criteria

- Adolescents at high risk of imminent suicide as reported by the students themselves, or parents/primary caregivers, or identified by the trained assessment team (AT) during screening.
- Adolescents with acute medical conditions who require immediate or ongoing in-patient medical or psychiatric care, as reported by student themselves or parents/primary caregivers or identified by the trained AT during screening.
- Adolescents with deafness, blindness and speech difficulties or with a severe mental, neurological or substance use disorders (eg, psychosis, mutism, intellectual disability, autism or drug dependence) identified by the trained AT during screening.

### Sample size calculations

The cluster unit of randomisation has been defined at the school level, stratified by gender. Based on other school-based and community-based mental health interventions using the measures assessing psychosocial problems in children,^15 16^ we assume an effect size of 0.4 at 3 months’ postintervention follow-up, with 80% power, 0.05 significance, an ICC of 0.05 and a two-sided hypothesis test with 40 school clusters randomised with a 1:1 allocation ratio, stratified by gender and accounting for 20% attrition. This results in a total of 550 adolescents (ie, 225 in each arm) and about 14 adolescents from each school. Each high school has about 150 adolescents in grade 8 and 9, which is more than sufficient to meet our sample size requirement. Stratification by gender will minimise imbalance between groups by factors likely to be associated with our outcome and reduce the between-cluster variability, hence increasing the power of the study.

### Recruitment procedure

The participants were recruited between 2 November 2021 and 30 November 2021. Informed consent from head-teachers, parents and assent from children was sought by trained research team for their participation in screening for the research study and for participation into the enrolment of the research study. Since the current study is school based, the permission to conduct the study was obtained from the school education department. Following informed consents and assents forms for the screening phase, the self-rated PSC was administered by the trained AT to screen adolescents for psychosocial distress in a private location in school settings for maintaining confidentiality. Participants meeting the eligibility criteria were enrolled for the study.

### Intervention

#### EASE intervention

Developed by the WHO, EASE is a brief, group psychological intervention programme^11^ based on evidence-informed techniques that are empirically supported for young adolescents living with symptoms of internalising disorders.^17^ The intervention is comprised of 7 weekly group sessions lasting 90 min for the young adolescents and is accompanied by three group sessions for their caregivers, each lasting approximately 90 min. The young adolescent sessions involve the following empirically supported components: psychoeducation, problem-solving, stress management, behavioural activation and relapse prevention. The caregiver sessions involve psychoeducation, active listening, quality time, praise, caregiver self-care and relapse prevention. The adolescent sessions will be delivered on weekly basis, and the three caregiver sessions are delivered at the third, fifth and seventh weeks of the adolescent intervention. Home practice of the EASE strategies is encouraged between each session for both adolescents and caregivers. Each EASE session is delivered by one primary facilitator and a cofacilitator.

#### Training and supervision of non-specialist facilitators in EASE intervention

EASE delivery school counsellors will be graduates with little or no prior experience of delivering targeted psychological interventions. EASE school counsellors will be selected from these fresh graduate students (having a bachelor degree in psychology) based on interviews and successful delivery of practice cases (at least one group each) under close supervision.

EASE school counsellors will receive 8 days (80–90 hours) of training by the master trainers of the intervention. Intervention training includes education on adversity and its impact on mental health, basic counselling skills, training in managing distressed participants, delivering EASE, skills in group facilitation and facilitator self-care. Further, school counsellors will have conducted at least one practice EASE intervention group under close supervision. Only school counsellors assessed as being competent (see quality control below) will be recruited to deliver the EASE intervention in the trial phase.

#### Supervision

Weekly supervision will be provided to EASE school counsellors by an appropriately qualified and trained supervisor for EASE with a good understanding of the young adolescent project to ensure fidelity of guidance provided, and to support school counsellors; in turn, this supervisor will be supported by clinical supervisors who have been involved in the development or training of EASE on a weekly to fortnightly basis and via Skype. Supervision will involve structured discussion of difficulties encountered in delivering EASE, management of adverse events as well as self-care for the staff. Supervision also forms an integral part of continued training (eg, through role-plays and associated teaching methods).

#### Competency and fidelity

Before and after the training of potential EASE providers, competency of the delivery agents will be evaluated using an adapted version of the Enhancing Assessment of Common Therapeutic factors (ENACT) rating scale.^18^ The ENACT scale is an 18-item assessment for common factors in psychological treatments, including task-sharing initiatives with non-specialists across cultural settings. School counsellors will also record checklists for each session as a measure of self-rated fidelity. An EASE specific checklist and four items assessing facilitation skills will be used to assess treatment fidelity and competency in a random sample of 10%–15% of directly observed sessions for each school counsellor.

#### Wait-list control

The wait-list control participants will receive usual care for the duration of their enrolment in the study. They will receive EASE immediately after trial evaluation on the basis that the results of the study demonstrate positive findings.

#### Outcome measures

Outcome measures will be administered with the adolescents and their parents at baseline, 1-week and at 3 months’ postintervention delivery. Assessments will be conducted by the trained AT who will be blind to allocation status of the participants. See outcomes measures table 1 given below for the details. All outcome measures have been translated in Urdu and adapted to suit the local cultural context as part of the associated feasibility study (trial registration NCT04254393).^12^

**Table 1:** Schedule of assessments

Sr. no	Outcomes	Time points			
		Screening	Baseline	Immediate (1-week) postintervention follow-up	3 months postintervention follow-up
Primary outcome (child’s level)					
1.	PSC	X	X	X	X
Secondary outcomes					
At child’s level					
2.	RCADS		X	X	X
3.	Somatic symptoms		X	X	X
4.	SPSI-R		X	X	X
5.	PESS		X	X	X
6.	PHQ-9		X	X	X
7.	SWEMWBS		X	X	X
8.	PedsQL		X	X	X
9.	PaedS				X
10.	PSYCHLOPS-Kid		X	X	X
At caregiver’s level					
11.	PedsQL-family impact		X	X	X
12.	CSRI		X		X
At both child and caregivers’ levels					
13.	APS		X	X	X
14.	SUQ		X	X	X

APS, Alabama Parenting Scale; CSRI, Client Services Receipt Inventory; PaedS, Paediatric Self-Stigmatisation Scale; PedsQL, Parent-rated Paediatric Quality of Life; PESS, Perceived Emotional/Personal Support Scale; PHQ-9, Patient Health Questionnaire; PSC, Paediatric Symptoms Checklist; PSYCHLOPS-Kid, Perceived Psychosocial Profile; RCADS, Revised Children’s Anxiety and Depression Scale; SPSI-R, Social Problem-Solving Inventory-Revised Short Form; SUQ, Strategy Use Questionnaire; SWEMWBS, Short Warwick Edinburgh Mental Well-being Scale.

### Primary outcome

#### Psychosocial distress

The primary outcome in this study is change in the scores of adolescent psychosocial distress at 3-month postintervention. Adolescents’ psychosocial distress will be measured at baseline, immediate (1 week), and at 3 months postintervention delivery in both arms using the PSC.^19^ The youth version of PSC has 35 items and three subscales; Externalising, internalising and attention problems with cut-offs of 7, 5 and 7 respectively. Items are rated on a three-point Likert scale (0=never, 1=sometimes, 2=often). Total score is calculated by summing the responses of all items. The recommended cut-off for administering PSC for adolescents in a new setting is ≥28.^20^ The cut-off score of ≥28 has been validated in the same study setting previously.^9^

### Secondary outcomes

1. The Revised Children’s Anxiety and Depression Scale (RCADS). The RCADS-25^21^ is a 25-item scale that measures levels of anxiety and low mood. The scale has two subscales (total anxiety and total depression) and an overall score. All items assess the frequency of symptoms and are rated on a four-point Likert scale. Each item is scored as 0 (never), 1 (sometimes), 2 (often), or 3 (always). Response values for each subscale is summed for calculating raw summary score.
2. Somatic symptoms checklist. The somatic symptoms checklist will be used to measure stress management among adolescents. It consists of 10 items, rated on a three-point scale (0=not true, 1=somewhat true, 2=very true or often true) based on the occurrence of the symptoms. Total score is calculated by summing the responses of all items. Higher score indicates frequent occurrence of somatic symptoms.
3. The Social Problem-Solving Inventory-Revised Short Form^22^ is a self-reporting questionnaire with 25 items. It consists of five subscales with five items each. Two of these subscales, ‘positive problem orientation’ and ‘negative problem orientation’, assess functional and dysfunctional cognitive and emotional orientations towards solving problems. The three remaining subscales, ‘rational problem-solving’, ‘impulsivity-carelessness style’ and ‘avoidance style’, assess problem-solving skills and behavioural style. The total score of this scale varies between 0 and 20 points. Highest scores correspond to better social problem-solving abilities. Social Problem-Solving Inventory-Scale has been used with adolescents in various countries including India and Pakistan.^23^
4. The Perceived Emotional/Personal Support Scale^24^ assesses perceived emotional support. Respondents are instructed to list the gender and first initial of three important people in each of three relationship categories: family members, non-family adults and friends. Using a four-point scale (hardly at all to very much), respondents answer the following questions about each person listed: ‘How much do you talk to them about personal concerns?’ ‘How close do you feel to them?’ and ‘How satisfied are you with the help and support they give you?’ How much do they talk to you about their concerns? Three support variables are created by averaging all ratings for all persons listed within each relationship category: perceived support from family, non-family adults and peers. Scores range from 1 to 4.
5. Patient Health Questionnaire (PHQ-9). PHQ-9, adapted for adolescents^25^ will be used to assess depressive symptoms and severity among adolescents. Items are rated on a four-point Likert scale with 0=not at all, 1=several day, 2=more than half the days, 3=nearly every day. Total score is calculated by summing the responses of all items. Higher score indicates higher incidence of depressive symptoms. PHQ-9 total score for the nine items ranges from 0 to 27.
6. Short Warwick Edinburgh Mental Wellbeing Scale (SWEMWBS). SWEMWBS^26^ is a brief questionnaire designed to measure mental well-being of children and adolescents over the past 2 weeks. It consists of 7 items rated on ‘none of the time’ to ‘all of the time’. The SWEMWBS is scored by first summing the score for each of the seven items and then transforming the total raw scores to metric scores using the SWEMWBS conversion table. The scores range from 7 to 35 and higher scores indicate higher positive mental well-being.^27^
7. Parent-rated Paediatric Quality of Life (PedsQL). Parent-rated PedsQL^28^ will be used to measure child’s health related quality of life during the past month. The scale measures child’s quality of health on four subscales namely, physical functioning, emotional functioning, social functioning and school functioning. The items are rated on four points Likert scale ranging from^1^ ‘no problem’ to^4^ ‘almost always a problem’. Items are then reverse-scored and linearly transformed to a 0–100, so that higher scores indicate better quality of life. This tool yields a total score (of all 23 items) and domain scores including physical health summary score (eight items), psychosocial health summary score (10 items) and school functioning score (five items).
8. Paediatric Self-Stigmatisation Scale (PaedS). The PaedS, is a scale developed for measuring stigma in children and adolescents.^29^ It consists of four subscales that measure societal devaluation, personal rejection, self-stigma and secrecy of receiving mental health treatment. The personal rejection subscale (five items) of the PaedS will be used in this study. The items are rated on four-point Likert scale, where higher scores indicate greater stigmatisation. The tool has been validated for the content and previously used in Pakistan.^30^
9. Peds-QL-Family impact module. Peds-QL family impact module^31^ will be used to assess parents’ health related quality of life. PedsQL family impact module is a 36-item scale that measures quality of life on six subscales namely; physical functioning, emotional functioning, social functioning, cognitive functioning, communication, worry, daily activities and family relationships. Items are rated on a five-point Likert scale (0=never to 4=almost always). Total score is calculated by summing all 36 items divided by the number of items answered. Higher scores indicate better functioning (less negative impact).^28^
10. Alabama Parenting Scale. Alabama parenting scale^32^ will be used to measure parenting practices. Alabama parenting scale is a 42-item measure that encompasses five dimensions of parenting that are relevant to the aetiology and treatment of children’s’ and adolescents’ problems^1^: positive involvement with children,^2^ supervision and monitoring,^3^ use of positive discipline techniques,^4^ consistency in the use of such discipline and^5^ use of corporal punishment. Items are rated on a five-point Likert scale (1=never to 5=almost always). Total score is calculated by summing all items.^33^
11. Strategy Use Questionnaire (SUQ). SUQ^34^ is designed to measure the use of coping strategies (identifying emotions and using relaxation technique, behavioural activation, problem-solving at child and understanding child’s internalising problems, using active listening skill, spending quality time with children, punishing child or using unhealthy disciplinary strategies and using relaxation technique at caregivers). Each item is scored on a frequency scale ranging from 0 (never) to 4 (all of the time). Total score is calculated by summing all items.
12. Health Services Utilisation. The cost of health services utilisation from the time proceeding assessment will be assessed with the adapted Client Services Receipt Inventory (CSRI).^35^ It has been adapted to use for the families of children with psychosocial distress. It measures the utilisation of various health and social care services including time and opportunity losses by the families in the care of their child with psychosocial distress.

### Child’s psychosocial well-being and functioning (PSYCHLOPS)-Kids

Child’s insight into his/her problems and well-being will be measured using the self-administered PSYCHLOPS-Kids.^36^ The outcome measure assesses three domains, including problems, functioning and well-being. PSYCHLOPS KIDS has three questionnaires forms, that is, pretherapy, during therapy and post therapy version. The tool is designed to be user friendly and can be used for children as young as 7 years. The tool has been feasibility tested as part of pilot study.

### Randomisation and blinding

The unit of randomisation is schools, which will be stratified by gender. Forty middle and high schools nominated by district education authority have been enrolled in the cRCT. Schools will be randomised on 1:1 allocation ratio by independent researcher using computerised software. The 40 schools will be randomised into intervention (n=20) and control arm (n=20). A total of 550 adolescents with psychosocial distress will be recruited from all randomised schools (10–14 adolescents from each cluster). Allocation concealment will be ensured by keeping the random assignments in sequentially numbered sealed envelopes. Due to the nature of the intervention, it is not possible to blind parents, adolescents, school counsellors, intervention supervisors, data and trial manager to the treatment allocation status of trial participants. The AT and principal investigators (PIs) will be blind to the allocation status of school clusters, while the qualitative research team will be un-blind to allocation status of school clusters. To maintain blinding during the trial, intervention and AT will not have any interaction during the trial by being based at separate office locations. The AT will also be non-residents of study subdistrict, Gujar Khan. Furthermore, participants including parents, school administration, head teachers and adolescents will each be individually instructed not to disclose to the AT which type of training they are receiving prior to the commencement of any assessment. Fidelity of blinding will be measured by having assessors guess the condition of each participant at the end of each assessment. We hypothesise that assessors will only be able to correctly guess the condition of participants at a chance rate of nearly 50% at follow-up assessments, indicating that blinding is maintained. The trial statistician will also be blind to the allocation status when developing the statistical analysis plan and writing the statistical programmes. The statistical analysis plan will be validated using dummy randomisation codes. The allocation status of research participants will only be disclosed in a trial steering committee (TSC) meeting after locking of the database on the completion of the trial. In the event of un-blinding, the point of unblinding will be recorded, the assessment will be halted and another assessor will be assigned to complete assessments for that cluster.

### Safety measures

Throughout the study, participants will have access to mental health experts at the IoP. When necessary, they will be referred to a mental health specialist for further assessment for any identified child protection issues or management of severe psychiatric problems including suicidal behaviour. School staff and students in the public-sector schools are entitled to healthcare in public dispensaries and hospitals through an existing referral system. Individuals are able to seek specialist services as walk-in patients and through referrals from primary and secondary healthcare centres. Travel time from the study area to the specialist mental healthcare facility is about 1 hour using public transport. Free of cost ambulance services will be made available in case of a need for referral from PHC centres to the specialist facility. At the specialist facility waiting times are minimal (1 hour at the most) and services are available free of cost. Adolescents at-risk of social protection issues are provided appropriate care through collaboration between the district education department and department of social welfare. We will have additional safeguards that will strengthen these mechanisms and we will initiate the appropriate referral to these local authorities via the IoP should the need arise for health and protection concerns in participants.

The face-to-face contact with the trained school counsellor will ensure that psychological distress is monitored each week, and a measure of distress conducted preintervention and postintervention will be used to supplement this. School counsellors will be well trained to monitor for sudden changes in mood and potential suicidality, including training in how to respond to suicidality with the individual. All staff will be trained in following appropriate referral procedures, via the supervisor and trial coordinator, should a concern for suicide or child protection concern arise.

### Adverse events reporting

A critical incident register will be maintained during the study to record serious adverse events and other adverse events. Recognition and management of all serious adverse events and other adverse events will be included in the training of school counsellors and the research staff. This will include a series of steps which have to be followed once such an event occurs including their detection, classification, reporting requirements and mitigation. Adverse events will be reported to trial ethics committees.

### Data management

All quantitative research data including baseline and follow-up outcome assessment from the research participants will be collected by trained assessors electronically using tablet computers on an android based application named Open Data Kit (ODK). All research data will be remotely uploaded as a Comma Separated Values (CSV) file on the main data server running online using ODK by AT on each day of assessment. The data server will be Good Clinical Practices (GCP) compliant including a date and time stamp for original data entry; an audit trail documenting any subsequent changes will be maintained. Data will be exported from the web server at the end of each day and will be checked for consistency and quality by the data manager. Exported data sets will be stored in password protected computer, only accessible by the data manager. Database will be backed up on a daily basis and the back-up data will be stored on secure hard drives that will be stored in a secure location. Process monitoring data such as supervision attendance, number of supervision meetings conducted and data of number of intervention strategies implemented will be collected in paper form, and this will be manually entered and stored as CSV files. All quantitative research data will be deidentified.

The qualitative data will be collected in audio and paper formats and will be stored in locked filling cabinets. It will be manually entered for analysis by separating the identifying information such as participants’ names and school name from the main data. Identifiable data will never be stored on portable devices unless those devices are stored in secure physical storage. Appropriate firewalls, encryption and password protection will be used for network-connected devices used to store study data. Qualitative data will be recorded on digital recorders with the informed consent of participants, and at the end of each day audio files will be transferred to computers and deleted from portable devices, and stored in a password-protected folders on computers that are backed up daily. The audio files will be destroyed after transcription.

### Statistical analysis

**Figure 1:**
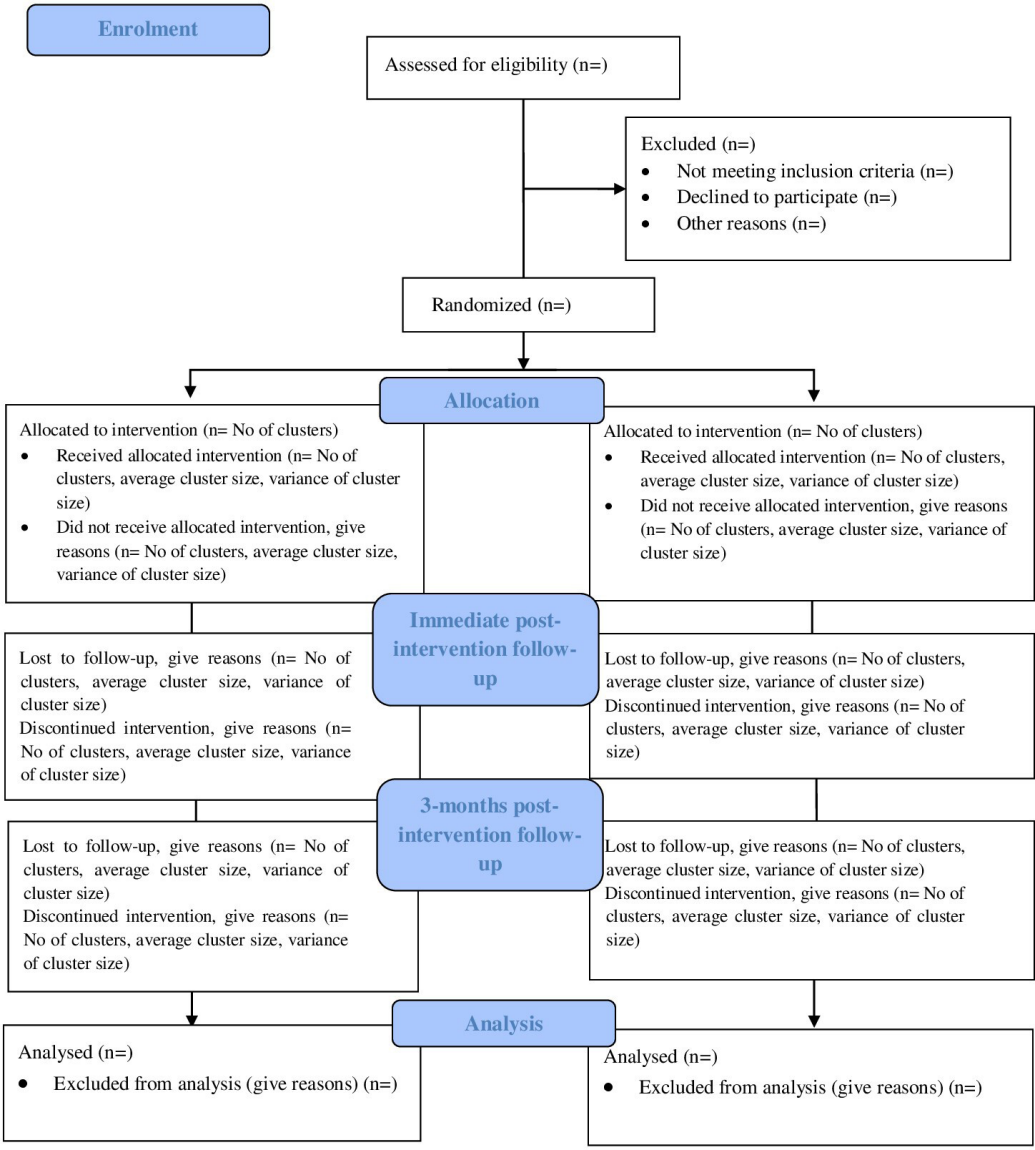
Flow of participants through cRCT

The findings of the trial will be reported following the updated recommendations of the Consolidated Standards of Reporting Trials (CONSORT) 2010 statement: extension to cluster randomised trials.^37^ This will include the flow of clusters and research participants through each stage of the trial, including the number eligible, randomly assigned, receiving the intended treatment, completing the study protocol and analysed for the primary outcome. Initial analyses will compare baseline characteristics of research participants across the two study arms; participants who complete follow-up assessments and participants who could not complete follow-up assessments; and a comparison of the distribution of potential confounding factors. The outcome measures will be summarised at baseline and 3-month post-intervention follow-up by intervention arm and overall. These will be summarised by means (SD), medians (IQR) or numbers and proportions as appropriate (and including age, gender, baseline outcome score), adjusting for cluster. Data will be cleaned and checked for accuracy prior to analysis. The CONSORT flow for the trial is given below (figure 1).

For the analysis of the primary outcome (reduction in PSC psychosocial distress scores from baseline to 3 months’ post intervention follow-up), a linear mixed model will be employed with treatment, visit, interaction between treatment and visit as fixed effects, gender and the baseline value of the PSC psychosocial distress score as covariates, subject and cluster (school cluster) as random effects. In addition, adjusted linear mixed model analysis will be performed with the pre-specified covariates (parent/primary caregivers’ education) measured at baseline being added into the above linear mixed model, which will be identified and listed in the statistical analysis plan. The crude and adjusted mean differences in the primary outcome together with its 95% CIs between intervention and control at 3 months will be derived from the mixed models. In addition, subgroup analysis of primary endpoint will be performed on the above pre-specified covariates. Analysis of secondary continuous outcomes with single follow-up measurement will be done using a linear mixed model with treatment as fixed effect, gender and the baseline value of the outcome variable as covariates and cluster (school cluster) as random effect. Analysis of secondary continuous outcomes with repeated follow-up measurement will be performed in a similar fashion as the primary endpoint analysis. The analysis of binary outcomes will use a generalised linear mixed model with treatment as fixed effect, baseline measurement of the outcome variable and gender as covariates, subject and cluster (school cluster) as random effects. The generalised linear mixed model will have a binomial distribution and logit link function, which will generate ORs with their 95% CIs of having an event between intervention and control.

Primary data analyses will be based on the intention-to-treat principle. The per-protocol analyses will also be performed as supplemental analysis. Descriptive statistics will be produced for outcome variables and also for baseline characteristics of participants by treatment arm and visit. Continuous variables will be summarised using number of observations, mean, median, SD, min and max by treatment arm and visit; categorical variables will be summarised by the number and percentage of research participants with mental health problems by treatment arm and visit. Adjusted analysis and subgroup analysis will be based on covariates at baseline without non-missing values.^38^ Detailed imputation methods will be described in the statistical analysis plan. All analyses will be detailed in the statistical analysis plan which will be finalised before the unblinding of the study. No interim analysis of outcomes is planned.

We will conduct a cost-effectiveness analysis to evaluate the cost-effectiveness of EASE intervention in improving outcomes. We will calculate service use for each participant using the data from CSRI.^35^ Service utilisation and the out-of-pocket expenditures of the participants (costs for seeing a doctor or other healthcare provider, admission to hospital, medicines, tests and extra help at home) will be collected at baseline and 3 months postintervention. The data collected through the CSRI will be used to calculate service costs and total costs of care for each participant. Unit costs of services itemised in the CSRI—such as cost per outpatient visit—will be based on locally conducted health facility costing exercises.

Service cost data will subsequently be linked to primary and secondary study outcomes, in particular internalising symptoms scores to assess issues around the value or cost-effectiveness of the EASE intervention. In the event that dominance is not shown, that is, the EASE intervention is more effective but the costs are also more than in the wait-list group, incremental cost-effectiveness ratios will be computed, together with their CIs (using bootstrapping techniques to overcome the expected skewness of the cost data). Results will be plotted on a cost-effectiveness plane and presented as cost-effectiveness acceptability curves to show the probability of the intervention being cost-effective at a range of willingness-to-pay threshold levels. A sensitivity analysis will be conducted to take account of uncertainty and imprecision in the measurements, including multiple imputation models for missing values.

### Qualitative process evaluation

Qualitative methods will be used to assess assumptions underlying the intervention strategy. In-depth interviews (IDIs) and focus group discussion (FGDs) will explore key programme implementation outcome variables and will cover intervention: acceptability, feasibility, appropriateness (including cultural appropriateness), fidelity, adoption and participants’ view about intervention’s perceived impact (both negative and positive) and ethics and safety concerns (Proctor, 2009). Following well-established procedures, qualitative interviews will be recorded, transcribed in Urdu and analysed in the original language (translation into English will only take place for the purpose of international reporting). IDIs and FGDs will be conducted at the preferred venue of the respondents, whether at home, at school or at any other place of convenience where privacy for IDIs/FDGs can be assured.

#### Sampling

Interviews will be conducted with both adolescents and caregivers in the intervention including completers and drop-outs, non-specialist facilitators, supervisory and school staff (teachers and head teachers). Sampling for qualitative interviews will be purposive based on the knowledge and exposure to EASE for each category of respondent. Sampling for IDIs will continue until theoretical saturation has been reached, anticipated to require 8–15 interviews with each category of respondent.

IDIs and FGDs will be conducted by the qualitative research team who will be trained in the key principles of qualitative interviewing. One interviewer will ask the questions and the other will take notes of the interview. Audio recordings will also be taken. All data will be anonymised and no identifying information will be collected during the interview. The interviews will follow a semistructured interview guide addressing topics relevant to each category of respondents (table 2).

**Table 2:** EASE semi structured interview summary guide

Sample	Themes
Non-specialist facilitators (delivery agents in EASE)	Intervention’s acceptability, feasibility, appropriateness (including cultural appropriateness), fidelity, adoption, intervention’s perceived impact (both negative and positive), ethics and safety concerns
Beneficiaries (adolescents in EASE)	Intervention’s acceptability, feasibility, appropriateness (including cultural appropriateness), adoption, intervention’s perceived impact (both negative and positive) and safety concerns
Beneficiaries (caregivers in EASE)	Intervention’s acceptability, feasibility, appropriateness (including cultural appropriateness), adoption, intervention’s perceived impact (both negative and positive) and safety concerns
Supervisory staff	Intervention’s acceptability, feasibility, appropriateness (including cultural appropriateness), fidelity, adoption, intervention’s perceived impact (both negative and positive), ethics and safety concerns
School staff	Barriers and facilitator of implementing intervention in school settings including perceived impact of intervention (both negative and positive)

EASE, Early Adolescent Skills for Emotions.

Analysis of IDIs/FGDs will be thematic, aided by application of the framework approach,^39^ which complements applied research, offers transparency in the analysis process and has an ability to move from descriptive narrative accounts to conceptual explanations. Analysis will move through phases of familiarisation, generation of codes and selection of illustrative quotes,^40^ conducted by multiple members of the qualitative research team. Data from FDGs and IDIs will be triangulated to ensure a comprehensive understanding through convergence or divergence of findings relating to each topic explored in interviews.

### Trial management

The TSC comprising PI, coinvestigator (s), trial coordinator, senior researchers and intervention staff who will meet monthly to oversight the study and manage the trial.

### Ethics

Ethical approval for the current study was obtained from Central Ethics Committee of University of Liverpool, UK, Ethics Review Committee of WHO Geneva and from Human Development Research Foundation Institutional Review Board, Islamabad, Pakistan. Data collection will be proceeded after seeking informed from the primary caregiver and assent from adolescents. All of the team members will be trained to ensure safety and confidentiality of participants throughout the research. An independent TSC will be set up to ensure human subject protection to the highest standards.

### Dissemination

The dissemination of intervention will be carried through peer reviewed publications and training to the relevant institutions to inform the Education and Health Ministries of Pakistan, in order to scale-up programme to public sector education. In addition to that the results will be disseminated through WHO’s media channels.^41^ The results of this study will be disseminated in Urdu to community key stakeholders (such as participant school head teachers, children and parents/primary caregivers) through reports or community presentations.

## Supplementary Material

Reviewer comments

Author's manuscript
