# Supplementary material for: Improving psychosocial distress for young adolescents in rural schools of Pakistan: study protocol of a cluster randomised controlled trial
Source: BMJ Open. 2022 Sep 23;12(9):e063607. doi: 10.1136/bmjopen-2022-063607 (PMC9511576; doi:10.1136/bmjopen-2022-063607)
Supplement: Author's manuscript [file bmjopen-2022-063607.draft_revisions.pdf]

BMJ Open

BMJ Open is committed to open peer review. As part of this commitment we make the peer review history of every article we publish publicly available.

When an article is published we post the peer reviewers' comments and the authors' responses online. We also post the versions of the paper that were used during peer review. These are the versions that the peer review comments apply to.

The versions of the paper that follow are the versions that were submitted during the peer review process. They are not the versions of record or the final published versions. They should not be cited or distributed as the published version of this manuscript.

BMJ Open is an open access journal and the full, final, typeset and author-corrected version of record of the manuscript is available on our site with no access controls, subscription charges or pay-per-view fees (http://bmjopen.bmj.com).

If you have any questions on BMJ Open's open peer review process please email info.bmjopen@bmj.com

BMJ Open

Improving psychosocial distress for young adolescents in rural schools of Pakistan: Study protocol of a cluster Randomized Controlled Trial

Journal:	*BMJ Open*
Manuscript ID	bmjopen-2022-063607
Article Type:	Protocol
Date Submitted by the Author:	05-Apr-2022

Complete List of Authors:	Hamdani, Syed Usman; Shifa Tameer-e-Millat University, Global Institute of Human Development; University of Liverpool Faculty of Health and Life Sciences, Department of Primary Care and Mental Health<br>Huma, Zill-e-; University of Liverpool Faculty of Health and Life Sciences, Department of Primary Care and Mental Health ; University of Liverpool Faculty of Health and Life Sciences, Department of Primary Care and Mental Health<br>Malik, Aiysha; World Health Organization, Mental Health and Substance Use<br>Nizami, Asad; Institute of Psychiatry<br>Baneen, Um ul; Human Development Research Foundation, Child and Adolescent Mental Health; Shifa Tameer-e-Millat University, Global Institute of Human Development<br>Suleman, Nadia; Human Development Research Foundation, Child and Adolescent Mental Health; Shifa Tameer-e-Millat University, Global Institute of Human Development<br>Javed, Hashim; Human Development Research Foundation, Child and Adolescent Mental Health; Shifa Tameer-e-Millat University, Global Institute of Human Development<br>Wang, Duolao; Liverpool School of Tropical Medicine, Department of Clinical Sciences<br>Van Ommeren, Mark; World Health Organization, Dep Mental Health and Substance Use<br>Mazhar, Samra; Pakistan Ministry of National Health Services Regulations and Coordination<br>Khan, Shahzad; World Health Organization<br>Minhas, Fareed; Institue of Psychiatry, Rawalpindi; Shifa Tameer-e-Millat University, Global Institute of Human Development<br>Rahman, Atif; University of Liverpool Faculty of Health and Life Sciences, Primary Care and Mental Health

Keywords:	Child & adolescent psychiatry < PSYCHIATRY, Depression & mood disorders < PSYCHIATRY, MENTAL HEALTH, Anxiety disorders < PSYCHIATRY

SCHOLARONE™
Manuscripts

Improving psychosocial distress for young adolescents in rural schools of Pakistan: Study protocol of a cluster Randomized Controlled Trial

Syed Usman Hamdani[1,2,3,5], Zill-e-Huma [1,2,3], Aiysha Malik[4], Asad Tamizuddin-Nizami[5], Um-ul-Baneen [1,3], Nadia Suleman [1,3], Hashim Javed[1,3], Duolao Wang[6], Mark van Ommeren[4], Samra Mazhar[7], Shahzad Alam Khan[8], Fareed Aslam Minhas[3], Atif Rahman[2]

Affiliations:

[1]Human Development Research Foundation (HDRF), Pakistan
[2]Department of Primary Care and Mental Health, University of Liverpool, UK
[3]Global Institute of Human Development, Shifa Tameer-e-Millat University, Pakistan
[4] Department of Mental Health and Substance Use, World Health Organization (WHO), Geneva, Switzerland
[5]Institute of Psychiatry, Rawalpindi Medical University, Benazir Bhutto Hospital, Rawalpindi, Pakistan
[6]Global Health Trials Unit, Liverpool School of Tropical Medicine, Liverpool, UK
[7]Ministry of National Health Services, Regulations and Coordination, Pakistan
[8]World Health Organization (WHO), Pakistan Office

Corresponding Author:
Syed Usman Hamdani
Global Institute of Human Development, Shifa Tameer-e-Millat, University, Islamabad, Pakistan
Email: syedusmanhamdani@gmail.com

For peer review only

For peer review only - http://bmjopen.bmj.com/site/about/guidelines.xhtml

Abstract

Introduction: Emotional problems are leading contributors to health burden among adolescents worldwide. There is an urgent need for evidence-based psychological interventions for young people. The present study aims to evaluate the effectiveness of a school-based, group psychological intervention (Early Adolescent Skills for Emotions [EASE]) developed by the World Health Organization (WHO) to improve psychosocial distress in Pakistani adolescents.

Method and analysis: A two-arm, single-blinded, cluster Randomized Controlled Trial (cRCT), with a wait-list control arm is being conducted in school settings of rural Pakistan. Forty eligible public-school clusters have been randomized (stratified by gender) on a 1:1 allocation ratio into intervention (n=20) and control arm (n=20). Following informed consent, 564 adolescents with psychosocial distress (Youth-reported Pediatric Symptoms Checklist [PSC], cut-off ≥ 28) from 40 schools have been enrolled into the trial (14±3 average cluster size) between 2nd to 30th November, 2021. Participants in the intervention arm will receive EASE in 7-weekly adolescents and 3-biweekly caregivers group sessions in schools. The adolescent sessions involve the components of psycho-education, stress management, behavioral activation, problem solving, and relapse prevention. Caregivers will receive training to learn and implement active listening; spending quality time and using praise as a strategy to help their children. The primary outcome is reduction in psychosocial distress at 3-months post-intervention. Secondary outcomes include symptoms of depression and anxiety, caregiver-adolescent relationship and caregivers' wellbeing. Outcomes will be assessed at baseline, immediate 1-week and 3-months-post-intervention. Qualitative process evaluation will explore barriers and facilitators to program implementation in low resource school settings.

Ethics: Ethics approval has been obtained from Central Ethics Committee of University of Liverpool, UK and Ethics Review Committee of WHO Geneva.

Dissemination: If the current study demonstrates effectiveness of the intervention, it will be disseminated by WHO and through peer-reviewed publications.

Trial registration number: ISRCTN17755448

Keywords: adolescents, psychosocial distress, public schools, Early Adolescents Skills for Emotions, non-specialist providers

For peer review only - http://bmjopen.bmj.com/site/about/guidelines.xhtml

Strengths and limitations of this study

- To help young adolescents with internalizing problems, a trans-diagnostic psychological intervention was developed by WHO called "Early Adolescent Skills for Emotions (EASE)". The intervention is designed to be delivered through non-specialist facilitators in low resource settings.
- A two arm, single blinded, cluster Randomized Controlled Trial (cRCT), with a wait-list control arm, adequate sample size and power and an embedded qualitative process evaluation is being conducted to evaluate the effectiveness and cost-effectiveness of EASE in school settings of Pakistan.
- The study is being conducted in one rural geographical area of Pakistan and may need more studies in other areas for generalizability.
- The study uses used self-reported measures for most outcomes. However, these are considered standard and used widely in the field.

For peer review only - http://bmjopen.bmj.com/site/about/guidelines.xhtml

87 **List of Abbreviations**

WHO	World Health Organization
EASE	Early Adolescent Skills for Emotions
cRCT	cluster Randomized Controlled Trial
PSC	Paediatric Symptom Checklist
PHQ-A	Patient Health Questionnaire for Adolescents
IDIs	In-Depth Interviews
FGDs	Focus Group Discussions
LMICs	Low and Middle-Income Countries
PM+	Problem Management Plus
PTSD	Post-Traumatic Stress Disorder
IoP-WCC	Institute of Psychiatry-WHO Collaborating Centre for mental health research and training
HDRF	Human Development Research Foundation.
CBT	Cognitive Behavioural Therapy
ENACT	ENhancing Assessment of Common Therapeutic factors
RCADS	Revised Children's Anxiety and Depression Scale
SWEWS	Short Warwick Edinburgh Mental Wellbeing Scale
PedsQL	Paediatric Quality of life
PaedS	Paediatric Self-Stigmatization Scale
CSRI	Client Service Receipt Inventory
PSYCHLOPS	Psychological Outcome Profiles
ODK	Open Data Kit
CSV	Comma Separated Values
AT	Assessment Team
GCP	Good Clinical Practice
CONSORT	Consolidated Standards of Reporting Trials
SD	Standard Deviation

For peer review only

For peer review only - http://bmjopen.bmj.com/site/about/guidelines.xhtml

IQR	Inter Quartile Range
TSC	Trial Steering Committee
mhGAP	Mental Health Gap Action Programme
UoL	University of Liverpool
NIHR	National Institutes of Health Research
DFID	Department for International Development
MRC	Medical Research Council

For peer review only

For peer review only - http://bmjopen.bmj.com/site/about/guidelines.xhtml

Introduction

Schools are an important public health platform to promote positive youth mental health globally (1, 2). Schools are uniquely placed to reach significant numbers of young people to address their mental health needs, especially in low- and middle-income countries (LMICs), where a lack of child and adolescent mental health services and experts; lack of access to mental health services; low awareness of mental health, and economic and societal barriers such as stigma remain key challenges to the provision of evidence-based mental health services (3). There is growing evidence from both high income and LMICs that school-based mental health programs are associated with beneficial mental health outcomes in adolescents (1). However, scale-up and sustainable implementation of mental health programs for young people in LMICs demands political will, stakeholder involvement, intersectoral coordination and leadership often between health and education sectors (4), particularly in a post-COVID context, where 91% of the world's student population has been negatively impacted by closures of schools due to COVID-19 pandemic (5).

Rates of anxiety and depression in young people have been exacerbated during COVID-19 pandemic (6). Moreover, due to exacerbation of psychosocial stressors including alteration in daily routine and social interactions during COVID-19 pandemic, at-risk adolescents are more likely to develop severe psychological problems. This situation demands an urgent need to provide more effective mental health support to school going adolescents to ensure that young people with symptoms of distress have access to the support that they need in their schools.

Youth in Pakistan account for 35% of its population (below the age of 14 years) and are exposed to a number of chronic adversities such as poverty, violence and socio and economic inequalities which makes them more susceptible to develop mental health problems early in their adolescence (7). In addition, the on-going COVID-19 pandemic situation is adversely affecting the economic, social and emotional wellbeing of the population at large and is particularly negatively impacting the wellbeing of adolescents due to nationwide lockdowns, closure of schools and disruption of academic year (8). A recent epidemiological study, conducted pre-COVID-19, with 5,856 adolescents from 41 public schools in rural Pakistan reported 25% prevalence rate of psychosocial distress in school going adolescents (9).

Recognizing the ever-growing burden of adolescent mental health problems in Pakistan, the Ministry of Health in Pakistan has launched the President's program to promote youth mental health through schools (10). It emphasizes the role of early-life interventions to promote mental health and prevent mental illnesses and takes a multi-tiered approach and recommends training non-specialists such as 'university graduates' to promote socio-emotional wellbeing of school-going adolescents. Under the ambit of President' Program, Early Adolescents Skills for Emotions (EASE) (*strategy 4 of the HAT toolkit*), is being implemented in public schools of rural Pakistan to address internalizing problems in school-going adolescents. EASE was developed by WHO as a brief, trans-diagnostic group psychological intervention designed to be delivered by non-specialists, to help young people aged approximately 10 to 14 years experiencing internalising problems such as psychosocial distress and symptoms of depression and anxiety (11) (*for more details on intervention please see section on interventions below*). EASE has been previously translated in Urdu (*National Language of Pakistan*), culturally adapted for implementation in public schools and feasibility tested in 8 public schools of rural Pakistan using a feasibility cluster randomized controlled trial design (trial registration NCT04254393). The results of the evaluation demonstrated the acceptability and feasibility of the adapted intervention to be delivered by non-

For peer review only - http://bmjopen.bmj.com/site/about/guidelines.xhtml

specialist facilitators in public school settings of Pakistan and exhibited promising effects on improving adolescent's wellbeing (*publication forthcoming*).

The current study aims to evaluate the effectiveness and cost-effectiveness of the culturally adapted EASE intervention compared to wait-list control to improve psychosocial distress in adolescents and improve caregiver-adolescent relationship and caregivers' wellbeing at 3-months post-intervention in public school settings of Pakistan.

Hypotheses

Our primary hypothesis is that *EASE* will be superior compared to *waitlist control*, in reducing the psychosocial distress in adolescents (aged 13-15 years), measured with the self-rated Paediatric Symptom Checklist at 3-months' post-intervention. Our secondary hypotheses are that *EASE* will result in improving adolescent wellbeing, quality of life, problem solving skills, perceived emotional support, caregiver-adolescents relationship and caregivers' wellbeing and reducing somatic complaints and anxiety and depressive symptoms in adolescents.

Methods and analysis

Study design

A two arm, single blind, cluster Randomized Controlled Trial (cRCT), with a wait-list control arm and an embedded qualitative study is being conducted in public schools of rural sub-district of Gujar Khan in Rawalpindi, Pakistan. The unit of randomization is a school. In the present study, 40 eligible school clusters, stratified by gender, have been randomized into intervention and control arms with a 1:1 allocation ratio. Outcomes will be assessed at baseline and post-intervention (1 week) and 3-months post-intervention.

Patient and public involvement

The research team has culturally adapted and feasibility tested the intervention by working collaboratively with the adolescents, caregivers and school administration from the same study sub-district. As a part of the formative phase we conducted a) qualitative needs assessments with school adolescents to identify the priority adolescent mental health problems; b) end-user testing workshops with adolescents in school settings to culturally adapt the EASE intervention and c) consultative workshops with relevant stakeholders from Ministries of education and health of Pakistan, school staff including head teachers, teachers, and mental health experts, parents and adolescents to develop a hypothesised pathway for the implementation of school based mental health programs in low resource settings of Pakistan (12). Once the current trial is complete, the findings will be disseminated to participants, ministries of health and education, school education department and wider public through presentations at community and public forums.

Study settings

The study will be conducted in 40 middle and high public schools of rural sub-district of Gujar Khan, located in the Rawalpindi district in the province of Punjab in Pakistan (approx. population of 1000 000). It is a pilot site for the implementation of President's Mental Health Program and falls under the catchment area of Institute of Psychiatry (IoP), Benazir Bhutto Hospital, Rawalpindi which is the sponsoring institute of the present study. The sub-district is semi-rural, with agrarian-based economy and represents typical rural area in the country. The population speaks Punjabi with Potohari being the predominant dialect. In Gujar Khan sub-district, there are 497 public schools (323-primary, 89 middle and 85 high schools). There are 231 schools for boys and 266 schools for girls in Gujar Khan in total. The primary decision body for public schools in Gujar Khan is District Education Department and with its permission 40 public schools (20 boys and 20 girls schools) were included in the current study. Literacy rates in the study district are 80% (13).

For peer review only - http://bmjopen.bmj.com/site/about/guidelines.xhtml

Mental health services in Pakistan are provided through specialist mental health units at tertiary healthcare facilities, concentred in urban centres with little or no mental health care for rural populations. School health services in public schools are provided through School Health and Nutrition Supervisors, who are based at Primary Health Care (PHC) centres and visit schools once a month to screen students for Eye, ENT, Dental, Skin and General Physical problems and if any health problems are identified, the students are referred to the medical officer of the concerned PHC centres.

Research participants

The age range of adolescents in school studying in academic grades 8-10 are 13-15 years of age. Our formative work indicated that challenges faced by adolescents in grades 8-10 include academic stress, expectations of high academic achievements from parents and teachers, peer pressure, interpersonal problems, worries about the future, and stressful home environment (publication forthcoming). These stressors often lead to mental health problems including distress, anxiety and depression like symptoms among adolescents. The need for focused psychological support for the mental health of adolescents in this age group has been identified as a priority by the education sector stakeholders.

The research participants for the current study are adolescents, screened positive for psychosocial distress with cut off score of ≥28 on self-rated Paediatric Symptoms Checklist (PSC) (validated cut-off score for school going adolescents in Pakistan (9).

Eligibility criteria of participants

Inclusion criteria

- Adolescents aged 13-15 years
- Living with parents/primary caregivers, attending public middle and high schools in the Gujar Khan sub-district of Rawalpindi, Pakistan.
- Written parent/primary caregiver informed consent or witnessed consent and adolescent assent for participation in the study.
- Screened positive on self-reported Paediatric Symptom Checklist (PSC) (cut- off score ≥ 28).
- Where there is more than one eligible child in a family unit, we will include the youngest eligible child.

Exclusion criteria

- Adolescents at high risk of imminent suicide as reported by the students themselves, or parents/primary caregivers, or identified by the trained assessment team during screening.
- Adolescents with acute medical conditions who require immediate or on-going in-patient medical or psychiatric care, as reported by student themselves or parents/primary caregivers or identified by the trained assessment team during screening.
- Adolescents with deafness, blindness and speech difficulties or with a severe mental, neurological or substance use disorders (e.g., psychosis, mutism, intellectual disability, autism, or drug dependence) identified by the trained assessment team during screening.

Sample Size calculations

The cluster unit of randomization has been defined at the school level, stratified by gender. Based on other school- and community-based mental health interventions using the measures assessing

For peer review only - http://bmjopen.bmj.com/site/about/guidelines.xhtml

psychosocial problems in children (14, 15), we assume an effect size of 0.4 at 3 months' post-intervention follow-up, with 80% power, 0.05 significance, an ICC of 0.05, and a two-sided hypothesis test with 40 school clusters randomised with a 1:1 allocation ratio, stratified by gender and accounting for 20% attrition. This results in a total of 550 adolescents (i.e., 225 in each arm) and about 14 adolescents from each school. Each high school has about 150 adolescents in grade 8 and 9, which is more than sufficient to meet our sample size requirement. Stratification by gender will minimize imbalance between groups by factors likely to be associated with our outcome and reduce the between-cluster variability, hence increasing the power of the study.

Recruitment Procedure

The participants were recruited between Nov 2nd and Nov 30th, 2021. Informed consent from head-teachers, parents and assent from children was sought by trained research team for their participation in screening for the research study and for participation into the enrolment of the research study. Since the current study is school-based, the permission to conduct the study was obtained from the school education department. Following informed consents and assents forms for the screening phase, the self-rated Paediatric Symptoms Checklist (PSC) was administered by the trained assessment team to screen adolescents for psychosocial distress in a private location in school settings for maintaining confidentiality. Participants meeting the eligibility criteria were enrolled for the study.

Intervention

Early Adolescent Skills for Emotions (EASE) Intervention

Developed by the WHO, EASE is a brief, group psychological intervention program (11) based on evidence-informed techniques that are empirically supported for young adolescents living with symptoms of internalising disorders (16). The intervention is comprised of 7 weekly group sessions lasting 90 minutes for the young adolescents and is accompanied by 3 group sessions for their caregivers, each lasting approximately 90 minutes. The young adolescent sessions involve the following empirically supported components: psychoeducation, problem solving, stress management, behavioural activation, and relapse prevention. The caregiver sessions involve psychoeducation, active listening, quality time, praise, caregiver self-care and relapse prevention. The adolescent sessions will be delivered on weekly basis, and the three caregiver sessions are delivered at the third, fifth and seventh weeks of the adolescent intervention. Home practice of the EASE strategies is encouraged between each session for both adolescents and caregivers. Each EASE session is delivered by one primary facilitator and a co-facilitator.

Training and supervision of non-specialist facilitators in 'EASE intervention

EASE delivery school counsellors will be graduates with little or no prior experience of delivering targeted psychological interventions. EASE school counsellors will be selected from these fresh graduate students (having a bachelor degree in Psychology) based upon interviews and successful delivery of practice cases (at least 1 group each) under close supervision.

EASE school counsellors will receive 8-days (80-90 hours) of training by the master trainers of the intervention. Intervention training includes education on adversity and its impact upon mental health, basic counselling skills, training in managing distressed participants, delivering EASE, skills in group facilitation, and facilitator self-care. Further, school counsellors will have conducted at least one practice EASE intervention group under close supervision. Only school counsellors assessed as being competent (see quality control below) will be recruited to deliver the EASE intervention in the trial phase.

Supervision

For peer review only - http://bmjopen.bmj.com/site/about/guidelines.xhtml

Weekly supervision will be provided to EASE school counsellors by an appropriately qualified and trained supervisor for EASE with a good understanding of the young adolescent project to ensure fidelity of guidance provided, and to support school counsellors; in turn, this supervisor will be supported by clinical supervisors who have been involved in the development or training of EASE on a weekly to fortnightly basis and via Skype. Supervision will involve structured discussion of difficulties encountered in delivering EASE, management of adverse events as well as self-care for the staff. Supervision also forms an integral part of continued training (e.g., through role-plays and associated teaching methods).

Competency and fidelity

Before and after the training of potential EASE providers, competency of the delivery agents will be evaluated using an adapted version of the Enhancing Assessment of Common Therapeutic factors (ENACT) rating scale (17). The ENACT scale is an 18-item assessment for common factors in psychological treatments, including task-sharing initiatives with non-specialists across cultural settings. School counsellors will also record checklists for each session as a measure of self-rated fidelity. An EASE specific checklist and four items assessing facilitation skills will be used to assess treatment fidelity and competency in a random sample of 10-15% of directly observed sessions for each school counsellor.

Wait-list control

The wait-list control participants will receive usual care for the duration of their enrolment in the study. They will receive EASE immediately after trial evaluation on the basis that the results of the study demonstrate positive findings.

Outcome measures

Outcome measures will be administered with the adolescents and their parents at post-intervention and at 3 -months' post-intervention delivery. Assessments will be conducted by the trained assessment team who will be blind to allocation status of the participants. See outcomes measures table 1 given below for the details. All outcome measures have been translated in Urdu and adapted to suit the local cultural context as part of the associated feasibility study (trial registration NCT04254393).

Primary outcome

Psychosocial distress: The primary outcome in the present study is change in the scores of adolescent psychosocial distress at 3-month post-intervention. Adolescents' psychosocial distress will be measured at baseline, immediate (1-week), and at 3-months post-intervention delivery in both arms using the Paediatric Symptom Checklist (PSC) (18). The youth version of PSC has 35 items and 3 subscales; Externalizing, internalizing and attention problems with cut-offs of 7, 5 and 7 respectively. Items are rated on a three-point Likert scale (0= *never*, 1=s*ometimes*, 2=*often*). Total score is calculated by summing the responses of all items. The recommended cut-off for administering Paediatric Symptom Checklist for adolescents in a new setting is ≥ 28 (19). The cut-off score of ≥ 28 has been validated in the same study setting previously (9).

For peer review only - http://bmjopen.bmj.com/site/about/guidelines.xhtml

Table 1: Schedule of assessments

Sr. no.	Outcomes	Timepoints			
		**Screening**	**Baseline**	**Immediate (1-week) post-intervention follow-up**	**3-months post-intervention follow-up**
Primary outcome (child's level)					
1.	PSC	X	X	X	X
Secondary outcomes					
At child's level					
2.	RCADS		X	X	X
3.	Somatic symptoms		X	X	X
4.	SPSI-R		X	X	X
5.	PESS		X	X	X
6.	PHQ-9		X	X	X
7.	SWEMWS		X	X	X
8.	PedsQL		X	X	X
9.	PaedS		X	X	X
10.	PSYCHLOPS-Kid		X	X	X
At caregiver's level					
11.	PedsQL-Family Impact		X	X	X
12.	CSRI		X		X
At both child and caregivers' levels					
13.	APS		X	X	X
14.	SUQ		X	X	X

PSC, Paediatric Symptoms Checklist; RCADS, Revised Children's Anxiety and Depression Scale; SPSI-R, Social Problem-Solving Inventory - Revised Short Form; PESS, Perceived Emotional/Personal Support Scale; PHQ-9, Patient Health Questionnaire; SWEMWS, Short Warwick Edinburgh Mental Wellbeing Scale; PedsQL, Parent-rated Paediatric Quality of Life; PaedS, Paediatric Self-Stigmatization Scale; PSYCHLOPS-Kid, Perceived Psychosocial Profile; CSRI, Client Services Receipt Inventory; APS, Alabama Parenting Scale; SUQ, Strategy Use Questionnaire

Secondary outcomes

1. The Revised Children's Anxiety and Depression Scale (RCADS)

The Revised Children's Anxiety and Depression Scale-25 (RCADS-25) (20) is a 25-item scale that measures levels of anxiety and low mood. The scale has two subscales (Total Anxiety and Total Depression) and an overall score. All items assess the frequency of symptoms and are rated

For peer review only - http://bmjopen.bmj.com/site/about/guidelines.xhtml

on a 4-point Likert scale. Each item is scored as 0 (*never*), 1 (*sometimes*), 2 (*often*), or 3 (*always*). Response values for each subscale is summed for calculating raw summary score.

2. Somatic symptoms checklist

The somatic symptoms checklist will be used to measure stress management among adolescents. It consists of 10 items, rated on a 3-point scale (0=*not true*, 1=*somewhat true*, 2= *very true or often true*) based on the occurrence of the symptoms. Total score is calculated by summing the responses of all items. Higher score indicates frequent occurrence of somatic symptoms.

2. The Social Problem-Solving Inventory - Revised Short Form (21) is a self-reporting questionnaire with 25 items. It consists of five subscales with five items each. Two of these subscales, 'positive problem orientation' and 'negative problem orientation', assess functional and dysfunctional cognitive and emotional orientations towards solving problems. The three remaining subscales, 'rational problem solving', 'impulsivity-carelessness style', and 'avoidance style', assess problem-solving skills and behavioural style. The total score of this scale varies between 0 and 20 points. Highest scores correspond to better social problem-solving abilities. Social Problem-Solving Inventory – Scale has been used with adolescents in various countries including India and Pakistan (22).

3. The **Perceived Emotional/Personal Support Scale** (23) assesses perceived emotional support. Respondents are instructed to list the gender and first initial of three important people in each of three relationship categories: family members, non-family adults, and friends. Using a four-point scale (*hardly at all* to *very much*), respondents answer the following questions about each person listed: "*How much do you talk to them about personal concerns*?" "*How close do you feel to them*?" and "*How satisfied are you with the help and support they give you*?" *How much do they talk to you about their concerns*? Three support variables are created by averaging all ratings for all persons listed within each relationship category: perceived support from family, non-family adults, and peers. Scores range from 1 to 4.

4. Patient Health Questionnaire (PHQ-9)

PHQ-9, adapted for adolescents (24) will be used to assess depressive symptoms and severity among adolescents. Items are rated on a 4-point Likert scale with 0= *not at all*, 1= *several day*, 2= *more than half the days*, 3= *nearly every day*. Total score is calculated by summing the responses of all items. Higher score indicates higher incidence of depressive symptoms. PHQ-9 total score for the nine items ranges from 0 to 27.

5. Short Warwick Edinburgh Mental Wellbeing Scale (SWEMWBS)

SWEMWBS (25) is a brief questionnaire designed to measure mental wellbeing of children and adolescents over the past two weeks. It consists of 7 items rated on '*none of the time*' to '*all of the time*'. The SWEMWBS is scored by first summing the score for each of the seven items and then transforming the total raw scores to metric scores using the SWEMWBS conversion table. The scores range from 7 to 35 and higher scores indicate higher positive mental well-being (26).

6. Parent-rated Paediatric Quality of Life (PedsQL)

Parent-rated Paediatric Quality of Life (PedsQL) (27) will be used to measure child's health related quality of life during the past month. The scale measures child´s quality of health on four subscales namely, physical functioning, emotional functioning, social functioning and school functioning. The items are rated on 4 points Likert scale ranging from (1) '*no problem*' to (4) '*almost always a problem*'. Items are then reverse-scored and linearly transformed to a 0–100, so that higher scores indicate better quality of life. This tool yields a total score (of all 23 items) and domain scores

For peer review only - http://bmjopen.bmj.com/site/about/guidelines.xhtml

including physical health summary score (8 items), psychosocial health summary score (10 items) and school functioning score (5 items).

7. Paediatric Self-Stigmatization Scale (PaedS)

The PaedS, is a scale developed for measuring stigma in children and adolescents (28). It consists of 4 subscales that measure societal devaluation, personal rejection, self-stigma and secrecy of receiving mental health treatment. The personal rejection subscale (5 items) of the PaedS will be used in the current study. The items are rated on 4-point Likert scale, where higher scores indicate greater stigmatization. The tool has been validated for the content and previously used in Pakistan (29).

8. Paediatric Quality of Life (Peds-QL)-Family impact module

Peds-QL family impact module (30) will be used to assess parents' health related quality of life. PedsQL family impact module is a 36-item scale that measures quality of life on 6 sub-scales namely; physical functioning, emotional functioning, social functioning, cognitive functioning, communication, worry, daily activities and family relationships. Items are rated on a 5-point Likert scale (0 = *never* to 4 = *almost always*). Total score is calculated by summing all 36 items divided by the number of items answered. Higher scores indicate better functioning (less negative impact). (27).

9. Alabama Parenting Scale

Alabama parenting scale (31) will be used to measure parenting practices. Alabama parenting scale is a 42-item measure that encompasses five dimensions of parenting that are relevant to the aetiology and treatment of children's' and adolescents' problems: (1) positive involvement with children, (2) supervision and monitoring, (3) use of positive discipline techniques, (4) consistency in the use of such discipline and (5) use of corporal punishment. Items are rated on a 5-point Likert scale (1 = never to 5 = almost always). Total score is calculated by summing all items. (32).

11. Strategy Use Questionnaire (SUQ) Strategy Use Questionnaire (SUQ) (33) is designed to measure the use of coping strategies (identifying emotions and using relaxation technique, behavioural activation, problem solving at child and understanding child's internalizing problems, using active listening skill, spending quality time with children, punishing child or using unhealthy disciplinary strategies and using relaxation technique at caregivers). Each item is scored on a frequency scale ranging from 0 (*never*) to 4 (*all of the time*). Total score is calculated by summing all items.

11. Health Services Utilization

The cost of health services utilization from the time proceeding assessment will be assessed with the adapted Client Services Receipt Inventory (CSRI) (34). It has been adapted to use for the families of children with psychosocial distress. It measures the utilization of various health and social care services including time and opportunity losses by the families in the care of their child with psychosocial distress.

Child's psycho-social wellbeing and functioning (PSYCHLOPS)-Kids

Child's insight into his/her problems and wellbeing will be measured using the self-administered PSYCHLOPS-Kids (35). The outcome measure assesses three domains, including problems, functioning and well-being. PSYCHLOPS KIDS has three questionnaires forms i.e., pre- therapy, during therapy and post therapy version. The tool is designed to be user friendly and can be used for children as young as 7 years. The tool has been feasibility tested as part of pilot study.

For peer review only - http://bmjopen.bmj.com/site/about/guidelines.xhtml

Randomization and blinding

The unit of randomization is schools, which will be stratified by gender. 40 middle and high schools nominated by district education authority have been enrolled in the cRCT. Schools will be randomized on 1:1 allocation ratio by independent researcher using computerized software. The 40 schools will be randomized into intervention (n=20) and control arm (n=20). 550 adolescents with psychosocial distress will be recruited from all randomized schools (10-14 adolescents from each cluster). Allocation concealment will be ensured by keeping the random assignments in sequentially numbered sealed envelopes. Due to the nature of the intervention, it is not possible to blind parents, adolescents, school counsellors, intervention supervisors, data and trial manager to the treatment allocation status of trial participants. The assessment team and PIs will be blind to the allocation status of school clusters, while the qualitative research team will be un-blind to allocation status of school clusters. To maintain blinding during the trial, intervention and assessment teams will not have any interaction during the trial by being based at separate office locations. The assessment team will also be non-residents of study sub-district, Gujar Khan. Furthermore, participants including parents, school administration, head teachers, and adolescents will each be individually instructed not to disclose to the assessment team which type of training they are receiving prior to the commencement of any assessment. Fidelity of blinding will be measured by having assessors guess the condition of each participant at the end of each assessment. We hypothesize that assessors will only be able to correctly guess the condition of participants at a chance rate of nearly 50% at follow-up assessments, indicating that blinding is maintained. The trial statistician will also be blind to the allocation status when developing the statistical analysis plan and writing the statistical programs. The statistical analysis plan will be validated using dummy randomization codes. The allocation status of research participants will only be disclosed in a trial steering committee meeting after locking of the database on the completion of the trial. In the event of un-blinding, the point of un-blinding will be recorded, the assessment will be halted, and another assessor will be assigned to complete assessments for that cluster.

Safety measures

Throughout the study, participants will have access to mental health experts at the IoP. When necessary, they will be referred to a mental health specialist for further assessment for any identified child protection issues or management of severe psychiatric problems including suicidal behaviour. School staff and students in the public-sector schools are entitled to health care in public dispensaries and hospitals through an existing referral system. Individuals are able to seek specialist services as walk-in patients and through referrals from primary and secondary health care centres. Travel time from the study area to the specialist mental health care facility is about 1 hour using public transport. Free of cost ambulance services will be made available in case of a need for referral from primary health care centres to the specialist facility. At the specialist facility waiting times are minimal (1 hour at the most) and services are available free of cost. Adolescents at-risk of social protection issues are provided appropriate care through collaboration between the district education department and department of social welfare. We will have additional safeguards that will strengthen these mechanisms and we will initiate the appropriate referral to these local authorities via the IoP should the need arise for health and protection concerns in participants.

The face-to-face contact with the trained school counsellor will ensure that psychological distress is monitored each week, and a measure of distress conducted pre- and post-intervention will be used to supplement this. School counsellors will be well trained to monitor for sudden changes in mood and potential suicidality, including training in how to respond to suicidality with the

For peer review only - http://bmjopen.bmj.com/site/about/guidelines.xhtml

individual. All staff will be trained in following appropriate referral procedures, via the supervisor and trial coordinator, should a concern for suicide or child protection concern arise.

Adverse events reporting

A critical incident register will be maintained during the study to record serious adverse events and other adverse events. Recognition and management of all serious adverse events and other adverse events will be included in the training of school counsellors and the research staff. This will include a series of steps which have to be followed once such an event occurs including their detection, classification, reporting requirements and mitigation. Adverse events will be reported to trial ethics committees.

Data management

All quantitative research data including baseline and follow-up outcome assessment from the research participants will be collected by trained assessors electronically using tablet computers on an android based application named Open Data Kit (ODK). All research data will be remotely uploaded as a Comma Separated Values (CSV) file on the main data server running online using ODK by Assessment Team (AT) on each day of assessment. The data server will be GCP compliant including a date- and time-stamp for original data entry; an audit trail documenting any subsequent changes will be maintained. Data will be exported from the web server at the end of each day and will be checked for consistency and quality by the data manager. Exported data sets will be stored in password protected computer, only accessible by the data manager. Database will be backed-up on a daily basis and the back-up data will be stored on secure hard drives that will be stored in a secure location. Process monitoring data such as supervision attendance, number of supervision meetings conducted and data of number of intervention strategies implemented will be collected in paper form, and this will be manually entered and stored as CSV files. All quantitative research data will be deidentified.

The qualitative data will be collected in audio and paper formats and will be stored in locked filling cabinets. It will be manually entered for analysis by separating the identifying information such as participants' names and school name from the main data. Identifiable data will never be stored on portable devices unless those devices are stored in secure physical storage. Appropriate firewalls, encryption, and password protection will be used for network-connected devices used to store study data. Qualitative data will be recorded on digital recorders with the informed consent of participants, and at the end of each day audio files will be transferred to computers and deleted from portable devices, and stored in a password protected folders on computers that are backed-up daily. The audio files will be destroyed after transcription.

Statistical analysis

The findings of the trial will be reported following the updated recommendations of the CONSORT 2010 statement: extension to cluster randomized trials (36). This will include the flow of clusters and research participants through each stage of the trial, including the number eligible, randomly assigned, receiving the intended treatment, completing the study protocol and analysed for the primary outcome. Initial analyses will compare baseline characteristics of research participants across the two study arms; participants who complete follow-up assessments and participants who could not complete follow up assessments; and a comparison of the distribution of potential confounding factors. The outcome measures will be summarized at baseline and 3-month post-intervention follow-up by intervention arm and overall. These will be summarized by means (SD), medians (IQR) or numbers and proportions as appropriate (and including age, gender,

For peer review only - http://bmjopen.bmj.com/site/about/guidelines.xhtml

baseline outcome score), adjusting for cluster. Data will be cleaned and checked for accuracy prior to analysis. The consort flow for the trial is given below (Figure 1).

For the analysis of the primary outcome (reduction in PSC psychosocial distress scores from baseline to 3-months' post intervention follow-up), a linear mixed model will be employed with treatment, visit, interaction between treatment and visit as fixed effects, gender and the baseline value of the PSC psychosocial distress score as covariates, subject and cluster (school cluster) as random effects. In addition, adjusted linear mixed model analysis will be performed with the pre-specified covariates (parent/primary caregivers' education) measured at baseline being added into the above linear mixed model, which will be identified and listed in the statistical analysis plan. The crude and adjusted mean differences in the primary outcome together with its 95% confidence intervals between intervention and control at 3-months will be derived from the mixed models. In addition, subgroup analysis of primary endpoint will be performed on the above pre-specified covariates. Analysis of secondary continuous outcomes with single follow-up measurement will be done using a linear mixed model with treatment as fixed effect, gender and the baseline value of the outcome variable as covariates, and cluster (school cluster) as random effect. Analysis of secondary continuous outcomes with repeated follow-up measurement will be performed in a similar fashion as the primary endpoint analysis. The analysis of binary outcomes will use a generalized linear mixed model with treatment as fixed effect, baseline measurement of the outcome variable and gender as covariates, subject and cluster (school cluster) as random effects. The generalized linear mixed model will have a binomial distribution and logit link function, which will generate odds ratios with their 95% confidence intervals of having an event between intervention and control.

Primary data analyses will be based on the intention-to-treat principle. The per-protocol analyses will also be performed as supplemental analysis. Descriptive statistics will be produced for outcome variables and also for baseline characteristics of participants by treatment arm and visit. Continuous variables will be summarized using number of observations, mean, median, standard deviation, min and max by treatment arm and visit; categorical variables will be summarized by the number and percentage of research participants with mental health problems by treatment arm and visit. Adjusted analysis and subgroup analysis will be based on covariates at baseline without non-missing values (37). Detailed imputation methods will be described in the statistical analysis plan. All analyses will be detailed in the statistical analysis plan which will be finalized before the un-blinding of the study. No interim analysis of outcomes is planned.

We will conduct a cost-effectiveness analysis to evaluate the cost-effectiveness of EASE intervention in improving outcomes. We will calculate service use for each participant using the data from Client Services Receipt Inventory (CSRI) (34). Service utilisation and the out-of-pocket expenditures of the participants (costs for seeing a doctor or other health-care provider, admission to hospital, medicines, tests and extra help at home) will be collected at baseline and 3-months post-intervention. The data collected through the CSRI will be used to calculate service costs and total costs of care for each participant. Unit costs of services itemised in the CSRI – such as cost per outpatient visit – will be based on locally conducted health facility costing exercises.

Service cost data will subsequently be linked to primary and secondary study outcomes, in particular internalizing symptoms scores to assess issues around the value or cost-effectiveness of the EASE intervention. In the event that dominance is not shown, i.e., the EASE intervention is more effective but the costs are also more than in the wait-list group, incremental cost-effectiveness ratios will be computed, together with their confidence intervals (using bootstrapping techniques

For peer review only - http://bmjopen.bmj.com/site/about/guidelines.xhtml

to overcome the expected skewness of the cost data). Results will be plotted on a cost-effectiveness plane and presented as cost-effectiveness acceptability curves to show the probability of the intervention being cost-effective at a range of willingness-to-pay threshold levels. A sensitivity analysis will be conducted to take account of uncertainty and imprecision in the measurements, including multiple imputation models for missing values.

Qualitative process evaluation

Qualitative methods will be used to assess assumptions underlying the intervention strategy. In-Depth Interviews (IDIs) and Focus Group Discussion (FGDs) will explore key program implementation outcome variables and will cover intervention: acceptability, feasibility, appropriateness (including cultural appropriateness), fidelity, adoption and participants' view about intervention's perceived impact (both negative and positive) and ethics and safety concerns (Proctor, 2009). Following well-established procedures, qualitative interviews will be recorded, transcribed in Urdu and analysed in the original language (translation into English will only take place for the purpose of international reporting). IDIs and FGDs will be conducted at the preferred venue of the respondents, whether at home, at school, or at any other place of convenience where privacy for IDIs / FDGs can be assured.

Sampling: Interviews will be conducted with both adolescents and caregivers in the intervention including completers and drop outs, non-specialist facilitators, supervisory and school staff (teachers and head teachers). Sampling for qualitative interviews will be purposive based upon the knowledge and exposure to EASE for each category of respondent. Sampling for in-depth interviews will continue until theoretical saturation has been reached, anticipated to require 8 – 15 interviews with each category of respondent.

IDIs and FGDs will be conducted by the qualitative research team who will be trained in the key principles of qualitative interviewing. One interviewer will ask the questions and the other will take notes of the interview. Audio recordings will also be taken. All data will be anonymised and no identifying information will be collected during the interview. The interviews will follow a semi-structured interview guide addressing topics relevant to each category of respondents (Table 2).

Table 2: EASE semi structured interview summary guide

Sample	Themes
Non-specialist facilitators (delivery agents in EASE)	Intervention's acceptability, feasibility, appropriateness (including cultural appropriateness), fidelity, adoption, intervention's perceived impact (both negative and positive), ethics and safety concerns
Beneficiaries (adolescents in EASE)	Intervention's acceptability, feasibility, appropriateness (including cultural appropriateness), adoption, intervention's perceived impact (both negative and positive) and safety concerns
Beneficiaries (caregivers in EASE)	Intervention's acceptability, feasibility, appropriateness (including cultural appropriateness), adoption, intervention's perceived impact (both negative and positive) and safety concerns
Supervisory staff	Intervention's acceptability, feasibility, appropriateness (including cultural appropriateness), fidelity, adoption, intervention's perceived impact (both negative and positive), ethics and safety concerns
School staff	Barriers and facilitator of implementing intervention in school settings including perceived impact of intervention (both negative and positive)

For peer review only - http://bmjopen.bmj.com/site/about/guidelines.xhtml

Analysis of in-depth interviews will be thematic, aided by application of the framework approach (38), which complements applied research, offers transparency in the analysis process, and has an ability to move from descriptive narrative accounts to conceptual explanations. Analysis will move through phases of familiarisation, generation of codes, and selection of illustrative quotes (39), conducted by multiple members of the qualitative research team. Data from FDGs and IDIs will be triangulated to ensure a comprehensive understanding through convergence or divergence of findings relating to each topic explored in interviews.

Trial management

The Trial Steering Committee (TSC) comprising of Principal Investigator (PI), Co-Investigator (s), trial coordinator, senior researchers and intervention staff who will meet monthly to oversight the study and manage the trial.

Ethics

Ethical approval for the current study was obtained from Central Ethics Committee of University of Liverpool, UK, Ethics Review Committee of World Health Organization (WHO) Geneva and from Human Development Research Foundation Institutional Review Board, Islamabad, Pakistan. Data collection will be proceeded after seeking informed from the primary caregiver and assent from adolescents. All of the team members will be trained to ensure safety and confidentiality of participants throughout the research. An independent Trial Steering Committee (TSC) will be set-up to ensure human subject protection to the highest standards.

Dissemination

The dissemination of intervention will be carried through peer reviewed publications and training to the relevant institutions to inform the Education and Health Ministries of Pakistan, in order to scale-up program to public sector education. In addition to that the results will be disseminated through WHO's media channels (40). The results of this study will be disseminated in Urdu to community key stakeholders (such as participant school head teachers, children and parents/primary caregivers) through reports or community presentations.

Acknowledgment

The study sponsor and funders have no role in the study design; collection, management, analysis, and interpretation of data; writing of the report; and the decision to submit the report for publication.

Authors' contributions: AR (Principal Investigator) conceived the study and was involved in developing the research design and supervising all aspects of the study. SUH (Co-Principal Investigator) was involved in conception of the study, developing the research design, study protocol, finalising the study tools, training and day-to-day supervision of the study team in Pakistan and drafted the initial manuscript. DW (Co-Investigator) was responsible for all the statistical aspects of the study including development of the statistical analysis plan. ZeH (trial coordinator) worked with UH to develop the research design, study protocol, finalising the study tools, training and day-to-day supervision of the study team and drafted the manuscript. AM, ATM, UB, NS, HJ, MvO, SM, SAK and FAM contributed to the writing.
All authors read and approved the final manuscript.

For peer review only - http://bmjopen.bmj.com/site/about/guidelines.xhtml

Funding: The project is co-funded by the Medical Research Council (MRC) and the Department of Health Social Care (DHSC) through its National Institutes of Health Research (NIHR) via grant award Reference MR/R022410/1.

Competing interests: None declared.

References

1. Fazel M, Hoagwood K, Stephan S, Ford T. Mental health interventions in schools 1: Mental health interventions in schools in high-income countries. The lancet Psychiatry. 2014;1(5):377-87.
2. Fazel M, Patel V, Thomas S, Tol W. Mental health interventions in schools in low-income and middle-income countries. The lancet Psychiatry. 2014;1(5):388-98.
3. Barry MM, Clarke AM, Jenkins R, Patel V. A systematic review of the effectiveness of mental health promotion interventions for young people in low and middle income countries. BMC public health. 2013;13:835.
4. Kumar D. School mental health program in India: Need to shift from a piecemeal approach to a long-term comprehensive approach with strong intersectoral coordination. Indian J Psychiatry. 2021;63(1):91-6.
5. Lee J. Mental health effects of school closures during COVID-19. The Lancet Child & adolescent health. 2020;4(6):421.
6. Jones EAK, Mitra AK, Bhuiyan AR. Impact of COVID-19 on Mental Health in Adolescents: A Systematic Review. Int J Environ Res Public Health. 2021;18(5):2470.
7. Hamdani SU, Huma ZE, Tamizuddin-Nizami A. Debate: Child and adolescent mental health services in Pakistan; Do we need in-patient mental health facilities for children and young people? Child and adolescent mental health. 2021;26(2):182-3.
8. Mian AI, Chachar AS. Debate: COVID-19 and school mental health in Pakistan. Child and adolescent mental health. 2020;25(4):270-2.
9. Hamdani SU, Huma ZE, Javed H, Warraitch A, Rahman A, Tameezuddin Nizami A, et al. Prevalence of psychosocial distress in school going adolescents in rural Pakistan: findings from a cross-sectional epidemiological survey. BJPsych Open. 2021;7(S1):S56-S7.
10. Mirza Z, Rahman A. Mental health care in Pakistan boosted by the highest office. Lancet (London, England). 2019;394(10216):2239-40.
11. Dawson KS, Watts S, Carswell K, Shehadeh MH, Jordans MJ, Bryant RA, et al. Improving access to evidence-based interventions for young adolescents: Early Adolescent Skills for Emotions (EASE). World Psychiatry. 2019;18(1):105.
12. Hamdani SU, Huma ZE, Suleman N, Warraitch A, Muzzafar N, Farzeen M, et al. Scaling-up school mental health services in low resource public schools of rural Pakistan: the Theory of Change (ToC) approach. International journal of mental health systems. 2021;15(1):8.
13. Population Council [Pakistan]. Landscape Analysis of Family Planning Situation in Pakistan: Brief Summary of Findings: District Population Council; 2016.
14. Durlak JA, Weissberg RP, Dymnicki AB, Taylor RD, Schellinger KB. The impact of enhancing students' social and emotional learning: A meta-analysis of school-based universal interventions. Child development. 2011;82(1):405-32.
15. Buchanan-Pascall S, Gray KM, Gordon M, Melvin GA. Systematic review and meta-analysis of parent group interventions for primary school children aged 4–12 years with externalizing and/or internalizing problems. Child Psychiatry & Human Development. 2018;49(2):244-67.

For peer review only - http://bmjopen.bmj.com/site/about/guidelines.xhtml

16. Organization WH. World health statistics 2016: monitoring health for the SDGs sustainable development goals: World Health Organization; 2016.
17. Kohrt BA, Jordans MJ, Rai S, Shrestha P, Luitel NP, Ramaiya MK, et al. Therapist competence in global mental health: development of the ENhancing Assessment of Common Therapeutic factors (ENACT) rating scale. Behaviour research and therapy. 2015;69:11-21.
18. Jellinek MS, Murphy JM, Robinson J, Feins A, Lamb S, Fenton T. Pediatric Symptom Checklist: screening school-age children for psychosocial dysfunction. The Journal of pediatrics. 1988;112(2):201-9.
19. Y-PSC P. Pediatric Symptom Checklist. Journal of Developmental and Behavioral Pediatrics. 1994;15:191-7.
20. Trickey D. RCADS: The Revised Children's Anxiety and Depression Scale(RCADS) and-The Revised Children's Anxiety and Depression Scale–Parent Version (RCADS-P). Guide to Using Outcomes and Feedback Tools. 2014:116.
21. D'Zurilla TJ, Nezu AM, Maydeu-Olivares A. Social problem-solving inventory-revised. 2002.
22. Chaudhry N, Rasool G. A case study on improving problem solving skills of undergraduate computer science students. World Applied Sciences Journal. 2012;20(1):34-9.
23. Slavin LA. Validation Studies of the PEPSS, a Measure of Perceived Emotional Support for Use with Adolescents. Journal of Adolescent Research. 1991;6(3):316-35.
24. Richardson LP, McCauley E, Grossman DC, McCarty CA, Richards J, Russo JE, et al. Evaluation of the Patient Health Questionnaire-9 Item for detecting major depression among adolescents. Journal of Pediatrics. 2010;126(6):1117-23.
25. Tennant R, Hiller L, Fishwick R, Platt S, Joseph S, Weich S, et al. The Warwick-Edinburgh mental well-being scale (WEMWBS): development and UK validation. Journal of Health and Quality of Life Outcomes. 2007;5(1):63.
26. Vaingankar JA, Abdin E, Chong SA, Sambasivam R, Seow E, Jeyagurunathan A, et al. Psychometric properties of the short Warwick Edinburgh mental well-being scale (SWEMWBS) in service users with schizophrenia, depression and anxiety spectrum disorders. Health and quality of life outcomes. 2017;15(1):1-11.
27. Varni JW, Seid M, Rode CA. The PedsQL™: measurement model for the pediatric quality of life inventory. Medical care. 1999:126-39.
28. Moses T. Self-labeling and its effects among adolescents diagnosed with mental disorders. Social science & medicine. 2009;68(3):570-8.
29. Khalil A, Gondal F, Imran N, Azeem MW. Self-Stigmatization in children receiving mental health treatment in Lahore, Pakistan. Asian journal of psychiatry. 2020;47:101839.
30. Varni JW, Seid M, Rode CA. The PedsQL™: measurement model for the pediatric quality of life inventory. Journal of Medical Care. 1999:126-39.
31. Zlomke KR, Lamport D, Bauman S, Garland B, Talbot B. Parenting Adolescents: Examining the Factor Structure of the Alabama Parenting Questionnaire for Adolescents. Journal of Child and Family Studies. 2014;23(8):1484-90.
32. Elgar FJ, Waschbusch DA, Dadds MR, Sigvaldason N. Development and validation of a short form of the Alabama Parenting Questionnaire. Journal of Child and Family Studies. 2007;16(2):243-59.
33. Brown FL, Steen F, Taha K, Aoun M, Bryant RA, Jordans MJD, et al. Early Adolescent Skills for Emotions (EASE) intervention for the treatment of psychological distress in adolescents: study protocol for randomised controlled trials in Lebanon and Jordan. Trials. 2019;20(1):545.

For peer review only - http://bmjopen.bmj.com/site/about/guidelines.xhtml

34. Chisholm D, Knapp MRJ, Knudsen HC, Amaddeo F, Gaite L, Van Wijngaarden B, et al. Client socio-demographic and service receipt inventory–European version: development of an instrument for international research: EPSILON Study 5. The British Journal of Psychiatry. 2000;177(S39):s28-s33.
35. Godfrey E, Aubrey M, Crockford S, Haythorne D, Kordowicz M, Ashworth M. The development and testing of PSYCHLOPS Kids: a new child-centred outcome measure. Child and adolescent mental health. 2019;24(1):54-65.
36. Campbell MK, Piaggio G, Elbourne DR, Altman DG. Consort 2010 statement: extension to cluster randomised trials. Bmj. 2012;345.
37. Enders C. Applied missing data analysis. 2010. New York: GuilfordGoogle Scholar.
38. Furber C. Framework analysis: a method for analysing qualitative data. African Journal of Midwifery and Women's health. 2010;4(2):97-100.
39. Green J, Thorogood N. Qualitative methods for health research: sage; 2018.
40. WHO. mhGAP intervention guide for mental, neurological and substance use disorders in non-specialized health settings: mental health Gap Action Programme (mhGAP): World Health Organization; 2016.

For peer review only

Figure 1: Flow of participants through cRCT

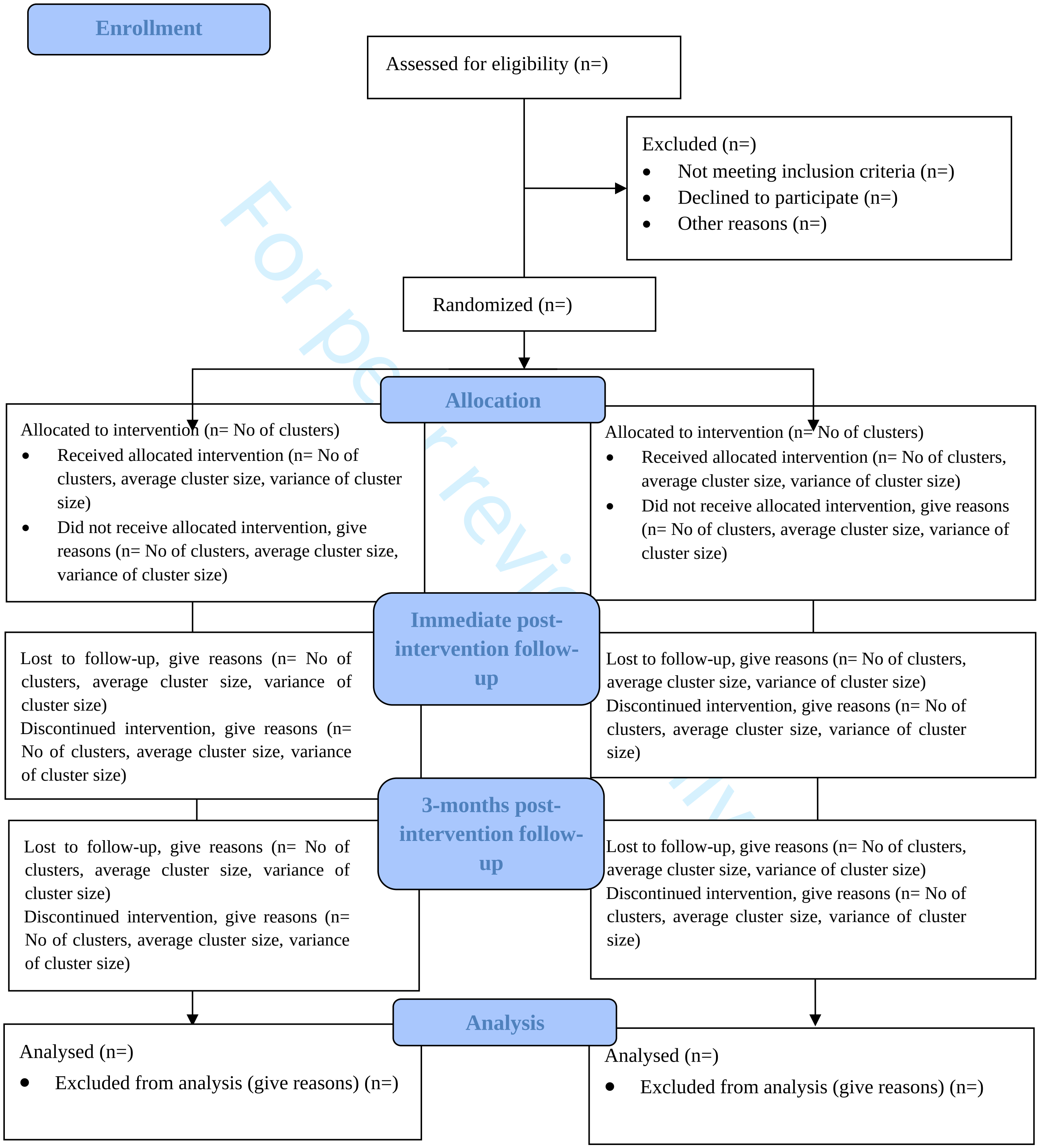

STANDARD PROTOCOL ITEMS: RECOMMENDATIONS FOR INTERVENTIONAL TRIALS

SPIRIT 2013 Checklist: Recommended items to address in a clinical trial protocol and related documents*

Section/item	Item No	Description	Addressed on page number
**Administrative information**			
Title	1	Descriptive title identifying the study design, population, interventions, and, if applicable, trial acronym	Title p.1
Trial registration	2a	Trial identifier and registry name. If not yet registered, name of intended registry	Abstract p. 2
	2b	All items from the World Health Organization Trial Registration Data Set	Included in the registration
Protocol version	3	Date and version identifier	N/A
Funding	4	Sources and types of financial, material, and other support	19
Roles and responsibilities	5a	Names, affiliations, and roles of protocol contributors	1 & 18
	5b	Name and contact information for the trial sponsor	N/A
	5c	Role of study sponsor and funders, if any, in study design; collection, management, analysis, and interpretation of data; writing of the report; and the decision to submit the report for publication, including whether they will have ultimate authority over any of these activities	18
	5d	Composition, roles, and responsibilities of the coordinating centre, steering committee, endpoint adjudication committee, data management team, and other individuals or groups overseeing the trial, if applicable (see Item 21a for data monitoring committee)	15

For peer review only

For peer review only - http://bmjopen.bmj.com/site/about/guidelines.xhtml

**Introduction**			
Background and rationale	6a	Description of research question and justification for undertaking the trial, including summary of relevant studies (published and unpublished) examining benefits and harms for each intervention	6-7
	6b	Explanation for choice of comparator	10
Objectives	7	Specific objectives or hypotheses	7
Trial design	8	Description of trial design including type of trial (eg, parallel group, crossover, factorial, single group), allocation ratio, and framework (eg, superiority, equivalence, noninferiority, exploratory)	7 & 14
**Methods: Participants, interventions, and outcomes**			
Study setting	9	Description of study settings (e.g., community clinic, academic hospital) and list of countries where data will be collected. Reference to where list of study sites can be obtained	7&8
Eligibility criteria	10	Inclusion and exclusion criteria for participants. If applicable, eligibility criteria for study centres and individuals who will perform the interventions (eg, surgeons, psychotherapists)	8
Interventions	11a	Interventions for each group with sufficient detail to allow replication, including how and when they will be administered	9-10
	11b	Criteria for discontinuing or modifying allocated interventions for a given trial participant (eg, drug dose change in response to harms, participant request, or improving/worsening disease)	N/A
	11c	Strategies to improve adherence to intervention protocols, and any procedures for monitoring adherence (eg, drug tablet return, laboratory tests)	N/A
	11d	Relevant concomitant care and interventions that are permitted or prohibited during the trial	10
Outcomes	12	Primary, secondary, and other outcomes, including the specific measurement variable (eg, systolic blood pressure), analysis metric (eg, change from baseline, final value, time to event), method of aggregation (eg, median, proportion), and time point for each outcome. Explanation of the clinical relevance of chosen efficacy and harm outcomes is strongly recommended	10-13
Participant timeline	13	Time schedule of enrolment, interventions (including any run-ins and washouts), assessments, and visits for participants. A schematic diagram is highly recommended (see Figure)	11

For peer review only - http://bmjopen.bmj.com/site/about/guidelines.xhtml

Sample size	14	Estimated number of participants needed to achieve study objectives and how it was determined, including clinical and statistical assumptions supporting any sample size calculations	8-9
Recruitment	15	Strategies for achieving adequate participant enrolment to reach target sample size	9

Methods: Assignment of interventions (for controlled trials)

Allocation:

Sequence generation	16a	Method of generating the allocation sequence (e.g., computer-generated random numbers), and list of any factors for stratification. To reduce predictability of a random sequence, details of any planned restriction (eg, blocking) should be provided in a separate document that is unavailable to those who enrol participants or assign interventions	14
Allocation concealment mechanism	16b	Mechanism of implementing the allocation sequence (e.g., central telephone; sequentially numbered, opaque, sealed envelopes), describing any steps to conceal the sequence until interventions are assigned	14
Implementation	16c	Who will generate the allocation sequence, who will enrol participants, and who will assign participants to interventions	14
Blinding (masking)	17a	Who will be blinded after assignment to interventions (eg, trial participants, care providers, outcome assessors, data analysts), and how	14
	17b	If blinded, circumstances under which unblinding is permissible, and procedure for revealing a participant's allocated intervention during the trial	14

Methods: Data collection, management, and analysis

Data collection methods	18a	Plans for assessment and collection of outcome, baseline, and other trial data, including any related processes to promote data quality (eg, duplicate measurements, training of assessors) and a description of study instruments (eg, questionnaires, laboratory tests) along with their reliability and validity, if known. Reference to where data collection forms can be found, if not in the protocol	15&16
	18b	Plans to promote participant retention and complete follow-up, including list of any outcome data to be collected for participants who discontinue or deviate from intervention protocols	N/A

Data management	19	Plans for data entry, coding, security, and storage, including any related processes to promote data quality (eg, double data entry; range checks for data values). Reference to where details of data management procedures can be found, if not in the protocol	15&16
Statistical methods	20a	Statistical methods for analysing primary and secondary outcomes. Reference to where other details of the statistical analysis plan can be found, if not in the protocol	15-17
	20b	Methods for any additional analyses (eg, subgroup and adjusted analyses)	15-17
	20c	Definition of analysis population relating to protocol non-adherence (eg, as randomised analysis), and any statistical methods to handle missing data (eg, multiple imputation)	16-17
**Methods: Monitoring**			
Data monitoring	21a	Composition of data monitoring committee (DMC); summary of its role and reporting structure; statement of whether it is independent from the sponsor and competing interests; and reference to where further details about its charter can be found, if not in the protocol. Alternatively, an explanation of why a DMC is not needed	18-19
	21b	Description of any interim analyses and stopping guidelines, including who will have access to these interim results and make the final decision to terminate the trial	N/A
Harms	22	Plans for collecting, assessing, reporting, and managing solicited and spontaneously reported adverse events and other unintended effects of trial interventions or trial conduct	15
Auditing	23	Frequency and procedures for auditing trial conduct, if any, and whether the process will be independent from investigators and the sponsor	N/A
**Ethics and dissemination**			
Research ethics approval	24	Plans for seeking research ethics committee/institutional review board (REC/IRB) approval	18
Protocol amendments	25	Plans for communicating important protocol modifications (eg, changes to eligibility criteria, outcomes, analyses) to relevant parties (eg, investigators, REC/IRBs, trial participants, trial registries, journals, regulators)	N/A

For peer review only - http://bmjopen.bmj.com/site/about/guidelines.xhtml

Consent or assent	26a	Who will obtain informed consent or assent from potential trial participants or authorised surrogates, and how (see Item 32)	9
	26b	Additional consent provisions for collection and use of participant data and biological specimens in ancillary studies, if applicable	N/A
Confidentiality	27	How personal information about potential and enrolled participants will be collected, shared, and maintained in order to protect confidentiality before, during, and after the trial	15
Declaration of interests	28	Financial and other competing interests for principal investigators for the overall trial and each study site	19
Access to data	29	Statement of who will have access to the final trial dataset, and disclosure of contractual agreements that limit such access for investigators	18
Ancillary and post-trial care	30	Provisions, if any, for ancillary and post-trial care, and for compensation to those who suffer harm from trial participation	N/A
Dissemination policy	31a	Plans for investigators and sponsor to communicate trial results to participants, healthcare professionals, the public, and other relevant groups (eg, via publication, reporting in results databases, or other data sharing arrangements), including any publication restrictions	18
	31b	Authorship eligibility guidelines and any intended use of professional writers	N/A
	31c	Plans, if any, for granting public access to the full protocol, participant-level dataset, and statistical code	N/A
**Appendices**			
Informed consent materials	32	Model consent form and other related documentation given to participants and authorised surrogates	N/A
Biological specimens	33	Plans for collection, laboratory evaluation, and storage of biological specimens for genetic or molecular analysis in the current trial and for future use in ancillary studies, if applicable	N/A

*It is strongly recommended that this checklist be read in conjunction with the SPIRIT 2013 Explanation & Elaboration for important clarification on the items. Amendments to the protocol should be tracked and dated. The SPIRIT checklist is copyrighted by the SPIRIT Group under the Creative Commons "Attribution-NonCommercial-NoDerivs 3.0 Unported" license.

For peer review only - http://bmjopen.bmj.com/site/about/guidelines.xhtml

BMJ Open

Improving psychosocial distress for young adolescents in rural schools of Pakistan: Study protocol of a cluster Randomized Controlled Trial

Journal:	*BMJ Open*
Manuscript ID	bmjopen-2022-063607.R1
Article Type:	Protocol
Date Submitted by the Author:	11-Apr-2022
Complete List of Authors:	Hamdani, Syed Usman; Shifa Tameer-e-Millat University, Global Institute of Human Development; University of Liverpool Faculty of Health and Life Sciences, Department of Primary Care and Mental Health<br>Huma, Zill-e-; University of Liverpool Faculty of Health and Life Sciences, Department of Primary Care and Mental Health ; University of Liverpool Faculty of Health and Life Sciences, Department of Primary Care and Mental Health<br>Malik, Aiysha; World Health Organization, Mental Health and Substance Use<br>Nizami, Asad; Institute of Psychiatry<br>Baneen, Um ul; Human Development Research Foundation, Child and Adolescent Mental Health; Shifa Tameer-e-Millat University, Global Institute of Human Development<br>Suleman, Nadia; Human Development Research Foundation, Child and Adolescent Mental Health; Shifa Tameer-e-Millat University, Global Institute of Human Development<br>Javed, Hashim; Human Development Research Foundation, Child and Adolescent Mental Health; Shifa Tameer-e-Millat University, Global Institute of Human Development<br>Wang, Duolao; Liverpool School of Tropical Medicine, Department of Clinical Sciences<br>Van Ommeren, Mark; World Health Organization, Dep Mental Health and Substance Use<br>Mazhar, Samra; Pakistan Ministry of National Health Services Regulations and Coordination<br>Khan, Shahzad; World Health Organization<br>Minhas, Fareed; Institue of Psychiatry, Rawalpindi; Shifa Tameer-e-Millat University, Global Institute of Human Development<br>Rahman, Atif; University of Liverpool Faculty of Health and Life Sciences, Primary Care and Mental Health
**Primary Subject Heading**:	Public health
Secondary Subject Heading:	Mental health, Evidence based practice, Research methods
Keywords:	Child & adolescent psychiatry < PSYCHIATRY, Depression & mood disorders < PSYCHIATRY, MENTAL HEALTH, Anxiety disorders < PSYCHIATRY

SCHOLARONE™
Manuscripts

Improving psychosocial distress for young adolescents in rural schools of Pakistan: Study protocol of a cluster Randomized Controlled Trial

Syed Usman Hamdani[1,2,3,5], Zill-e-Huma [1,2,3], Aiysha Malik[4], Asad Tamizuddin-Nizami[5], Um-ul-Baneen [1,3], Nadia Suleman [1,3], Hashim Javed[1,3], Duolao Wang[6], Mark van Ommeren[4], Samra Mazhar[7], Shahzad Alam Khan[8], Fareed Aslam Minhas[3], Atif Rahman[2]

Affiliations:

[1]Human Development Research Foundation (HDRF), Pakistan

[2]Department of Primary Care and Mental Health, University of Liverpool, UK

[3]Global Institute of Human Development, Shifa Tameer-e-Millat University, Pakistan

[4] Department of Mental Health and Substance Use, World Health Organization (WHO), Geneva, Switzerland

[5]Institute of Psychiatry, Rawalpindi Medical University, Benazir Bhutto Hospital, Rawalpindi, Pakistan

[6]Global Health Trials Unit, Liverpool School of Tropical Medicine, Liverpool, UK

[7]Ministry of National Health Services, Regulations and Coordination, Pakistan

[8]World Health Organization (WHO), Pakistan Office

Corresponding Author:

Syed Usman Hamdani

Global Institute of Human Development, Shifa Tameer-e-Millat, University, Islamabad, Pakistan

Email: syedusmanhamdani@gmail.com

For peer review only

For peer review only - http://bmjopen.bmj.com/site/about/guidelines.xhtml

Abstract

Introduction: Emotional problems are leading contributors to health burden among adolescents worldwide. There is an urgent need for evidence-based psychological interventions for young people. The present study aims to evaluate the effectiveness of a school-based, group psychological intervention (Early Adolescent Skills for Emotions [EASE]) developed by the World Health Organization (WHO) to improve psychosocial distress in Pakistani adolescents.

Method and analysis: A two-arm, single-blinded, cluster Randomized Controlled Trial (cRCT), with a wait-list control arm is being conducted in school settings of rural Pakistan. Forty eligible public-school clusters have been randomized (stratified by gender) on a 1:1 allocation ratio into intervention (n=20) and control arm (n=20). Following informed consent, 564 adolescents with psychosocial distress (Youth-reported Pediatric Symptoms Checklist [PSC], cut-off ≥ 28) from 40 schools have been enrolled into the trial (14±3 average cluster size) between 2nd to 30th November, 2021. Participants in the intervention arm will receive EASE in 7-weekly adolescents and 3-biweekly caregivers group sessions in schools. The adolescent sessions involve the components of psycho-education, stress management, behavioral activation, problem solving, and relapse prevention. Caregivers will receive training to learn and implement active listening; spending quality time and using praise as a strategy to help their children. The primary outcome is reduction in psychosocial distress at 3-months post-intervention. Secondary outcomes include symptoms of depression and anxiety, caregiver-adolescent relationship and caregivers' wellbeing. Outcomes will be assessed at baseline, immediate 1-week and 3-months-post-intervention. Qualitative process evaluation will explore barriers and facilitators to program implementation in low resource school settings.

Ethics: Ethics approval has been obtained from Central Ethics Committee of University of Liverpool, UK, Ethics Review Committee of WHO Geneva and from the Institutional Review Board of Human Development Research Foundation (HDRF), Pakistan.

Dissemination: The findings of the study will be disseminated by WHO and through peer-reviewed publications.

Trial registration number: ISRCTN17755448

Keywords: adolescents, psychosocial distress, public schools, Early Adolescents Skills for Emotions, non-specialist providers

For peer review only - http://bmjopen.bmj.com/site/about/guidelines.xhtml

Strengths and limitations of this study

- To help young adolescents with internalizing problems, a trans-diagnostic psychological intervention was developed by WHO called "Early Adolescent Skills for Emotions (EASE)". The intervention is designed to be delivered through non-specialist facilitators in low resource settings.
- A two arm, single blinded, cluster Randomized Controlled Trial (cRCT), with a wait-list control arm, adequate sample size and power and an embedded qualitative process evaluation is being conducted to evaluate the effectiveness and cost-effectiveness of EASE in school settings of Pakistan.
- The study is being conducted in one rural geographical area of Pakistan and may need more studies in other areas for generalizability.
- The study uses used self-reported measures for most outcomes. However, these are considered standard and used widely in the field.

For peer review only - http://bmjopen.bmj.com/site/about/guidelines.xhtml

List of Abbreviations

WHO	World Health Organization
EASE	Early Adolescent Skills for Emotions
cRCT	cluster Randomized Controlled Trial
PSC	Paediatric Symptom Checklist
PHQ-A	Patient Health Questionnaire for Adolescents
IDIs	In-Depth Interviews
FGDs	Focus Group Discussions
LMICs	Low and Middle-Income Countries
PM+	Problem Management Plus
PTSD	Post-Traumatic Stress Disorder
IoP-WCC	Institute of Psychiatry-WHO Collaborating Centre for mental health research and training
HDRF	Human Development Research Foundation.
CBT	Cognitive Behavioural Therapy
ENACT	ENhancing Assessment of Common Therapeutic factors
RCADS	Revised Children's Anxiety and Depression Scale
SWEWS	Short Warwick Edinburgh Mental Wellbeing Scale
PedsQL	Paediatric Quality of life
PaedS	Paediatric Self-Stigmatization Scale
CSRI	Client Service Receipt Inventory
PSYCHLOPS	Psychological Outcome Profiles
ODK	Open Data Kit
CSV	Comma Separated Values
AT	Assessment Team
GCP	Good Clinical Practice
CONSORT	Consolidated Standards of Reporting Trials
SD	Standard Deviation

For peer review only

For peer review only - http://bmjopen.bmj.com/site/about/guidelines.xhtml

IQR	Inter Quartile Range
TSC	Trial Steering Committee
mhGAP	Mental Health Gap Action Programme
UoL	University of Liverpool
NIHR	National Institutes of Health Research
DFID	Department for International Development
MRC	Medical Research Council

For peer review only

For peer review only - http://bmjopen.bmj.com/site/about/guidelines.xhtml

Introduction

Schools are an important public health platform to promote positive youth mental health globally (1, 2). Schools are uniquely placed to reach significant numbers of young people to address their mental health needs, especially in low- and middle-income countries (LMICs), where a lack of child and adolescent mental health services and experts; lack of access to mental health services; low awareness of mental health, and economic and societal barriers such as stigma remain key challenges to the provision of evidence-based mental health services (3). There is growing evidence from both high income and LMICs that school-based mental health programs are associated with beneficial mental health outcomes in adolescents (1). However, scale-up and sustainable implementation of mental health programs for young people in LMICs demands political will, stakeholder involvement, intersectoral coordination and leadership often between health and education sectors (4), particularly in a post-COVID context, where 91% of the world's student population has been negatively impacted by closures of schools due to COVID-19 pandemic (5).

Rates of anxiety and depression in young people have been exacerbated during COVID-19 pandemic (6). Moreover, due to exacerbation of psychosocial stressors including alteration in daily routine and social interactions during COVID-19 pandemic, at-risk adolescents are more likely to develop severe psychological problems. This situation demands an urgent need to provide more effective mental health support to school going adolescents to ensure that young people with symptoms of distress have access to the support that they need in their schools.

Youth in Pakistan account for 35% of its population (below the age of 14 years) and are exposed to a number of chronic adversities such as poverty, violence and socio and economic inequalities which makes them more susceptible to develop mental health problems early in their adolescence (7). In addition, the on-going COVID-19 pandemic situation is adversely affecting the economic, social and emotional wellbeing of the population at large and is particularly negatively impacting the wellbeing of adolescents due to nationwide lockdowns, closure of schools and disruption of academic year (8). A recent epidemiological study, conducted pre-COVID-19, with 5,856 adolescents from 41 public schools in rural Pakistan reported 25% prevalence rate of psychosocial distress in school going adolescents (9).

Recognizing the ever-growing burden of adolescent mental health problems in Pakistan, the Ministry of Health in Pakistan has launched the President's program to promote youth mental health through schools (10). It emphasizes the role of early-life interventions to promote mental health and prevent mental illnesses and takes a multi-tiered approach and recommends training non-specialists such as 'university graduates' to promote socio-emotional wellbeing of school-going adolescents. Under the ambit of President' Program, Early Adolescents Skills for Emotions (EASE) (*strategy 4 of the HAT toolkit*), is being implemented in public schools of rural Pakistan to address internalizing problems in school-going adolescents. EASE was developed by WHO as a brief, trans-diagnostic group psychological intervention designed to be delivered by non-specialists, to help young people aged approximately 10 to 14 years experiencing internalising problems such as psychosocial distress and symptoms of depression and anxiety (11) (*for more details on intervention please see section on interventions below*). EASE has been previously translated in Urdu (*National Language of Pakistan*), culturally adapted for implementation in public schools and feasibility tested in 8 public schools of rural Pakistan using a feasibility cluster randomized controlled trial design (trial registration NCT04254393). The results of the evaluation demonstrated the acceptability and feasibility of the adapted intervention to be delivered by non-

For peer review only - http://bmjopen.bmj.com/site/about/guidelines.xhtml

specialist facilitators in public school settings of Pakistan and exhibited promising effects on improving adolescent's wellbeing (*publication forthcoming*).

The current study aims to evaluate the effectiveness and cost-effectiveness of the culturally adapted EASE intervention compared to wait-list control to improve psychosocial distress in adolescents and improve caregiver-adolescent relationship and caregivers' wellbeing at 3-months post-intervention in public school settings of Pakistan.

Hypotheses

Our primary hypothesis is that *EASE* will be superior compared to *waitlist control*, in reducing the psychosocial distress in adolescents (aged 13-15 years), measured with the self-rated Paediatric Symptom Checklist at 3-months' post-intervention. Our secondary hypotheses are that *EASE* will result in improving adolescent wellbeing, quality of life, problem solving skills, perceived emotional support, caregiver-adolescents relationship and caregivers' wellbeing and reducing somatic complaints and anxiety and depressive symptoms in adolescents.

Methods and analysis

Study design

A two arm, single blind, cluster Randomized Controlled Trial (cRCT), with a wait-list control arm and an embedded qualitative study is being conducted in public schools of rural sub-district of Gujar Khan in Rawalpindi, Pakistan. The unit of randomization is a school. In the present study, 40 eligible school clusters, stratified by gender, have been randomized into intervention and control arms with a 1:1 allocation ratio. Outcomes will be assessed at baseline and post-intervention (1 week) and 3-months post-intervention.

Patient and public involvement

The research team has culturally adapted and feasibility tested the intervention by working collaboratively with the adolescents, caregivers and school administration from the same study sub-district. As a part of the formative phase we conducted a) qualitative needs assessments with school adolescents to identify the priority adolescent mental health problems; b) end-user testing workshops with adolescents in school settings to culturally adapt the EASE intervention and c) consultative workshops with relevant stakeholders from Ministries of education and health of Pakistan, school staff including head teachers, teachers, and mental health experts, parents and adolescents to develop a hypothesised pathway for the implementation of school based mental health programs in low resource settings of Pakistan (12). Once the current trial is complete, the findings will be disseminated to participants, ministries of health and education, school education department and wider public through presentations at community and public forums.

Study settings

The study will be conducted in 40 middle and high public schools of rural sub-district of Gujar Khan, located in the Rawalpindi district in the province of Punjab in Pakistan (approx. population of 1000 000). It is a pilot site for the implementation of President's Mental Health Program and falls under the catchment area of Institute of Psychiatry (IoP), Benazir Bhutto Hospital, Rawalpindi which is the sponsoring institute of the present study. The sub-district is semi-rural, with agrarian-based economy and represents typical rural area in the country. The population speaks Punjabi with Potohari being the predominant dialect. In Gujar Khan sub-district, there are 497 public schools (323-primary, 89 middle and 85 high schools). There are 231 schools for boys and 266 schools for girls in Gujar Khan in total. The primary decision body for public schools in Gujar Khan is District Education Department and with its permission 40 public schools (20 boys and 20 girls schools) were included in the current study. Literacy rates in the study district are 80% (13).

For peer review only - http://bmjopen.bmj.com/site/about/guidelines.xhtml

Mental health services in Pakistan are provided through specialist mental health units at tertiary healthcare facilities, concentred in urban centres with little or no mental health care for rural populations. School health services in public schools are provided through School Health and Nutrition Supervisors, who are based at Primary Health Care (PHC) centres and visit schools once a month to screen students for Eye, ENT, Dental, Skin and General Physical problems and if any health problems are identified, the students are referred to the medical officer of the concerned PHC centres.

Research participants

The age range of adolescents in school studying in academic grades 8-10 are 13-15 years of age. Our formative work indicated that challenges faced by adolescents in grades 8-10 include academic stress, expectations of high academic achievements from parents and teachers, peer pressure, interpersonal problems, worries about the future, and stressful home environment (publication forthcoming). These stressors often lead to mental health problems including distress, anxiety and depression like symptoms among adolescents. The need for focused psychological support for the mental health of adolescents in this age group has been identified as a priority by the education sector stakeholders.

The research participants for the current study are adolescents, screened positive for psychosocial distress with cut off score of ≥28 on self-rated Paediatric Symptoms Checklist (PSC) (validated cut-off score for school going adolescents in Pakistan (9).

Eligibility criteria of participants

Inclusion criteria

- Adolescents aged 13-15 years
- Living with parents/primary caregivers, attending public middle and high schools in the Gujar Khan sub-district of Rawalpindi, Pakistan.
- Written parent/primary caregiver informed consent or witnessed consent and adolescent assent for participation in the study.
- Screened positive on self-reported Paediatric Symptom Checklist (PSC) (cut- off score ≥ 28).
- Where there is more than one eligible child in a family unit, we will include the youngest eligible child.

Exclusion criteria

- Adolescents at high risk of imminent suicide as reported by the students themselves, or parents/primary caregivers, or identified by the trained assessment team during screening.
- Adolescents with acute medical conditions who require immediate or on-going in-patient medical or psychiatric care, as reported by student themselves or parents/primary caregivers or identified by the trained assessment team during screening.
- Adolescents with deafness, blindness and speech difficulties or with a severe mental, neurological or substance use disorders (e.g., psychosis, mutism, intellectual disability, autism, or drug dependence) identified by the trained assessment team during screening.

Sample Size calculations

The cluster unit of randomization has been defined at the school level, stratified by gender. Based on other school- and community-based mental health interventions using the measures assessing

For peer review only - http://bmjopen.bmj.com/site/about/guidelines.xhtml

psychosocial problems in children (14, 15), we assume an effect size of 0.4 at 3 months' post-intervention follow-up, with 80% power, 0.05 significance, an ICC of 0.05, and a two-sided hypothesis test with 40 school clusters randomised with a 1:1 allocation ratio, stratified by gender and accounting for 20% attrition. This results in a total of 550 adolescents (i.e., 225 in each arm) and about 14 adolescents from each school. Each high school has about 150 adolescents in grade 8 and 9, which is more than sufficient to meet our sample size requirement. Stratification by gender will minimize imbalance between groups by factors likely to be associated with our outcome and reduce the between-cluster variability, hence increasing the power of the study.

Recruitment Procedure

The participants were recruited between Nov 2nd and Nov 30th, 2021. Informed consent from head-teachers, parents and assent from children was sought by trained research team for their participation in screening for the research study and for participation into the enrolment of the research study. Since the current study is school-based, the permission to conduct the study was obtained from the school education department. Following informed consents and assents forms for the screening phase, the self-rated Paediatric Symptoms Checklist (PSC) was administered by the trained assessment team to screen adolescents for psychosocial distress in a private location in school settings for maintaining confidentiality. Participants meeting the eligibility criteria were enrolled for the study.

Intervention

Early Adolescent Skills for Emotions (EASE) Intervention

Developed by the WHO, EASE is a brief, group psychological intervention program (11) based on evidence-informed techniques that are empirically supported for young adolescents living with symptoms of internalising disorders (16). The intervention is comprised of 7 weekly group sessions lasting 90 minutes for the young adolescents and is accompanied by 3 group sessions for their caregivers, each lasting approximately 90 minutes. The young adolescent sessions involve the following empirically supported components: psychoeducation, problem solving, stress management, behavioural activation, and relapse prevention. The caregiver sessions involve psychoeducation, active listening, quality time, praise, caregiver self-care and relapse prevention. The adolescent sessions will be delivered on weekly basis, and the three caregiver sessions are delivered at the third, fifth and seventh weeks of the adolescent intervention. Home practice of the EASE strategies is encouraged between each session for both adolescents and caregivers. Each EASE session is delivered by one primary facilitator and a co-facilitator.

Training and supervision of non-specialist facilitators in 'EASE intervention

EASE delivery school counsellors will be graduates with little or no prior experience of delivering targeted psychological interventions. EASE school counsellors will be selected from these fresh graduate students (having a bachelor degree in Psychology) based upon interviews and successful delivery of practice cases (at least 1 group each) under close supervision.

EASE school counsellors will receive 8-days (80-90 hours) of training by the master trainers of the intervention. Intervention training includes education on adversity and its impact upon mental health, basic counselling skills, training in managing distressed participants, delivering EASE, skills in group facilitation, and facilitator self-care. Further, school counsellors will have conducted at least one practice EASE intervention group under close supervision. Only school counsellors assessed as being competent (see quality control below) will be recruited to deliver the EASE intervention in the trial phase.

Supervision

For peer review only - http://bmjopen.bmj.com/site/about/guidelines.xhtml

Weekly supervision will be provided to EASE school counsellors by an appropriately qualified and trained supervisor for EASE with a good understanding of the young adolescent project to ensure fidelity of guidance provided, and to support school counsellors; in turn, this supervisor will be supported by clinical supervisors who have been involved in the development or training of EASE on a weekly to fortnightly basis and via Skype. Supervision will involve structured discussion of difficulties encountered in delivering EASE, management of adverse events as well as self-care for the staff. Supervision also forms an integral part of continued training (e.g., through role-plays and associated teaching methods).

Competency and fidelity

Before and after the training of potential EASE providers, competency of the delivery agents will be evaluated using an adapted version of the Enhancing Assessment of Common Therapeutic factors (ENACT) rating scale (17). The ENACT scale is an 18-item assessment for common factors in psychological treatments, including task-sharing initiatives with non-specialists across cultural settings. School counsellors will also record checklists for each session as a measure of self-rated fidelity. An EASE specific checklist and four items assessing facilitation skills will be used to assess treatment fidelity and competency in a random sample of 10-15% of directly observed sessions for each school counsellor.

Wait-list control

The wait-list control participants will receive usual care for the duration of their enrolment in the study. They will receive EASE immediately after trial evaluation on the basis that the results of the study demonstrate positive findings.

Outcome measures

Outcome measures will be administered with the adolescents and their parents at post-intervention and at 3 -months' post-intervention delivery. Assessments will be conducted by the trained assessment team who will be blind to allocation status of the participants. See outcomes measures table 1 given below for the details. All outcome measures have been translated in Urdu and adapted to suit the local cultural context as part of the associated feasibility study (trial registration NCT04254393).

Primary outcome

Psychosocial distress: The primary outcome in the present study is change in the scores of adolescent psychosocial distress at 3-month post-intervention. Adolescents' psychosocial distress will be measured at baseline, immediate (1-week), and at 3-months post-intervention delivery in both arms using the Paediatric Symptom Checklist (PSC) (18). The youth version of PSC has 35 items and 3 subscales; Externalizing, internalizing and attention problems with cut-offs of 7, 5 and 7 respectively. Items are rated on a three-point Likert scale (0= *never*, 1=s*ometimes*, 2=*often*). Total score is calculated by summing the responses of all items. The recommended cut-off for administering Paediatric Symptom Checklist for adolescents in a new setting is ≥ 28 (19). The cut-off score of ≥ 28 has been validated in the same study setting previously (9).

For peer review only - http://bmjopen.bmj.com/site/about/guidelines.xhtml

Table 1: Schedule of assessments

Sr. no.	Outcomes	Timepoints			
		**Screening**	**Baseline**	**Immediate (1-week) post-intervention follow-up**	**3-months post-intervention follow-up**
Primary outcome (child's level)					
1.	PSC	X	X	X	X
Secondary outcomes					
At child's level					
2.	RCADS		X	X	X
3.	Somatic symptoms		X	X	X
4.	SPSI-R		X	X	X
5.	PESS		X	X	X
6.	PHQ-9		X	X	X
7.	SWEMWS		X	X	X
8.	PedsQL		X	X	X
9.	PaedS		X	X	X
10.	PSYCHLOPS-Kid		X	X	X
At caregiver's level					
11.	PedsQL-Family Impact		X	X	X
12.	CSRI		X		X
At both child and caregivers' levels					
13.	APS		X	X	X
14.	SUQ		X	X	X

PSC, Paediatric Symptoms Checklist; RCADS, Revised Children's Anxiety and Depression Scale; SPSI-R, Social Problem-Solving Inventory - Revised Short Form; PESS, Perceived Emotional/Personal Support Scale; PHQ-9, Patient Health Questionnaire; SWEMWS, Short Warwick Edinburgh Mental Wellbeing Scale; PedsQL, Parent-rated Paediatric Quality of Life; PaedS, Paediatric Self-Stigmatization Scale; PSYCHLOPS-Kid, Perceived Psychosocial Profile; CSRI, Client Services Receipt Inventory; APS, Alabama Parenting Scale; SUQ, Strategy Use Questionnaire

Secondary outcomes

1. The Revised Children's Anxiety and Depression Scale (RCADS)

The Revised Children's Anxiety and Depression Scale-25 (RCADS-25) (20) is a 25-item scale that measures levels of anxiety and low mood. The scale has two subscales (Total Anxiety and Total Depression) and an overall score. All items assess the frequency of symptoms and are rated

For peer review only - http://bmjopen.bmj.com/site/about/guidelines.xhtml

on a 4-point Likert scale. Each item is scored as 0 (*never*), 1 (*sometimes*), 2 (*often*), or 3 (*always*). Response values for each subscale is summed for calculating raw summary score.

2. Somatic symptoms checklist

The somatic symptoms checklist will be used to measure stress management among adolescents. It consists of 10 items, rated on a 3-point scale (0=*not true*, 1=*somewhat true*, 2= *very true or often true*) based on the occurrence of the symptoms. Total score is calculated by summing the responses of all items. Higher score indicates frequent occurrence of somatic symptoms.

2. The Social Problem-Solving Inventory - Revised Short Form (21) is a self-reporting questionnaire with 25 items. It consists of five subscales with five items each. Two of these subscales, 'positive problem orientation' and 'negative problem orientation', assess functional and dysfunctional cognitive and emotional orientations towards solving problems. The three remaining subscales, 'rational problem solving', 'impulsivity-carelessness style', and 'avoidance style', assess problem-solving skills and behavioural style. The total score of this scale varies between 0 and 20 points. Highest scores correspond to better social problem-solving abilities. Social Problem-Solving Inventory – Scale has been used with adolescents in various countries including India and Pakistan (22).

3. The **Perceived Emotional/Personal Support Scale** (23) assesses perceived emotional support. Respondents are instructed to list the gender and first initial of three important people in each of three relationship categories: family members, non-family adults, and friends. Using a four-point scale (*hardly at all* to *very much*), respondents answer the following questions about each person listed: "*How much do you talk to them about personal concerns*?" "*How close do you feel to them*?" and "*How satisfied are you with the help and support they give you*?" *How much do they talk to you about their concerns*? Three support variables are created by averaging all ratings for all persons listed within each relationship category: perceived support from family, non-family adults, and peers. Scores range from 1 to 4.

4. Patient Health Questionnaire (PHQ-9)

PHQ-9, adapted for adolescents (24) will be used to assess depressive symptoms and severity among adolescents. Items are rated on a 4-point Likert scale with 0= *not at all*, 1= *several day*, 2= *more than half the days*, 3= *nearly every day*. Total score is calculated by summing the responses of all items. Higher score indicates higher incidence of depressive symptoms. PHQ-9 total score for the nine items ranges from 0 to 27.

5. Short Warwick Edinburgh Mental Wellbeing Scale (SWEMWBS)

SWEMWBS (25) is a brief questionnaire designed to measure mental wellbeing of children and adolescents over the past two weeks. It consists of 7 items rated on '*none of the time*' to '*all of the time*'. The SWEMWBS is scored by first summing the score for each of the seven items and then transforming the total raw scores to metric scores using the SWEMWBS conversion table. The scores range from 7 to 35 and higher scores indicate higher positive mental well-being (26).

6. Parent-rated Paediatric Quality of Life (PedsQL)

Parent-rated Paediatric Quality of Life (PedsQL) (27) will be used to measure child's health related quality of life during the past month. The scale measures child´s quality of health on four subscales namely, physical functioning, emotional functioning, social functioning and school functioning. The items are rated on 4 points Likert scale ranging from (1) '*no problem*' to (4) '*almost always a problem*'. Items are then reverse-scored and linearly transformed to a 0–100, so that higher scores indicate better quality of life. This tool yields a total score (of all 23 items) and domain scores

For peer review only - http://bmjopen.bmj.com/site/about/guidelines.xhtml

including physical health summary score (8 items), psychosocial health summary score (10 items) and school functioning score (5 items).

7. Paediatric Self-Stigmatization Scale (PaedS)

The PaedS, is a scale developed for measuring stigma in children and adolescents (28). It consists of 4 subscales that measure societal devaluation, personal rejection, self-stigma and secrecy of receiving mental health treatment. The personal rejection subscale (5 items) of the PaedS will be used in the current study. The items are rated on 4-point Likert scale, where higher scores indicate greater stigmatization. The tool has been validated for the content and previously used in Pakistan (29).

8. Paediatric Quality of Life (Peds-QL)-Family impact module

Peds-QL family impact module (30) will be used to assess parents' health related quality of life. PedsQL family impact module is a 36-item scale that measures quality of life on 6 sub-scales namely; physical functioning, emotional functioning, social functioning, cognitive functioning, communication, worry, daily activities and family relationships. Items are rated on a 5-point Likert scale (0 = *never* to 4 = *almost always*). Total score is calculated by summing all 36 items divided by the number of items answered. Higher scores indicate better functioning (less negative impact). (27).

9. Alabama Parenting Scale

Alabama parenting scale (31) will be used to measure parenting practices. Alabama parenting scale is a 42-item measure that encompasses five dimensions of parenting that are relevant to the aetiology and treatment of children's' and adolescents' problems: (1) positive involvement with children, (2) supervision and monitoring, (3) use of positive discipline techniques, (4) consistency in the use of such discipline and (5) use of corporal punishment. Items are rated on a 5-point Likert scale (1 = never to 5 = almost always). Total score is calculated by summing all items. (32).

11. Strategy Use Questionnaire (SUQ) Strategy Use Questionnaire (SUQ) (33) is designed to measure the use of coping strategies (identifying emotions and using relaxation technique, behavioural activation, problem solving at child and understanding child's internalizing problems, using active listening skill, spending quality time with children, punishing child or using unhealthy disciplinary strategies and using relaxation technique at caregivers). Each item is scored on a frequency scale ranging from 0 (*never*) to 4 (*all of the time*). Total score is calculated by summing all items.

11. Health Services Utilization

The cost of health services utilization from the time proceeding assessment will be assessed with the adapted Client Services Receipt Inventory (CSRI) (34). It has been adapted to use for the families of children with psychosocial distress. It measures the utilization of various health and social care services including time and opportunity losses by the families in the care of their child with psychosocial distress.

Child's psycho-social wellbeing and functioning (PSYCHLOPS)-Kids

Child's insight into his/her problems and wellbeing will be measured using the self-administered PSYCHLOPS-Kids (35). The outcome measure assesses three domains, including problems, functioning and well-being. PSYCHLOPS KIDS has three questionnaires forms i.e., pre- therapy, during therapy and post therapy version. The tool is designed to be user friendly and can be used for children as young as 7 years. The tool has been feasibility tested as part of pilot study.

For peer review only - http://bmjopen.bmj.com/site/about/guidelines.xhtml

Randomization and blinding

The unit of randomization is schools, which will be stratified by gender. 40 middle and high schools nominated by district education authority have been enrolled in the cRCT. Schools will be randomized on 1:1 allocation ratio by independent researcher using computerized software. The 40 schools will be randomized into intervention (n=20) and control arm (n=20). 550 adolescents with psychosocial distress will be recruited from all randomized schools (10-14 adolescents from each cluster). Allocation concealment will be ensured by keeping the random assignments in sequentially numbered sealed envelopes. Due to the nature of the intervention, it is not possible to blind parents, adolescents, school counsellors, intervention supervisors, data and trial manager to the treatment allocation status of trial participants. The assessment team and PIs will be blind to the allocation status of school clusters, while the qualitative research team will be un-blind to allocation status of school clusters. To maintain blinding during the trial, intervention and assessment teams will not have any interaction during the trial by being based at separate office locations. The assessment team will also be non-residents of study sub-district, Gujar Khan. Furthermore, participants including parents, school administration, head teachers, and adolescents will each be individually instructed not to disclose to the assessment team which type of training they are receiving prior to the commencement of any assessment. Fidelity of blinding will be measured by having assessors guess the condition of each participant at the end of each assessment. We hypothesize that assessors will only be able to correctly guess the condition of participants at a chance rate of nearly 50% at follow-up assessments, indicating that blinding is maintained. The trial statistician will also be blind to the allocation status when developing the statistical analysis plan and writing the statistical programs. The statistical analysis plan will be validated using dummy randomization codes. The allocation status of research participants will only be disclosed in a trial steering committee meeting after locking of the database on the completion of the trial. In the event of un-blinding, the point of un-blinding will be recorded, the assessment will be halted, and another assessor will be assigned to complete assessments for that cluster.

Safety measures

Throughout the study, participants will have access to mental health experts at the IoP. When necessary, they will be referred to a mental health specialist for further assessment for any identified child protection issues or management of severe psychiatric problems including suicidal behaviour. School staff and students in the public-sector schools are entitled to health care in public dispensaries and hospitals through an existing referral system. Individuals are able to seek specialist services as walk-in patients and through referrals from primary and secondary health care centres. Travel time from the study area to the specialist mental health care facility is about 1 hour using public transport. Free of cost ambulance services will be made available in case of a need for referral from primary health care centres to the specialist facility. At the specialist facility waiting times are minimal (1 hour at the most) and services are available free of cost. Adolescents at-risk of social protection issues are provided appropriate care through collaboration between the district education department and department of social welfare. We will have additional safeguards that will strengthen these mechanisms and we will initiate the appropriate referral to these local authorities via the IoP should the need arise for health and protection concerns in participants.

The face-to-face contact with the trained school counsellor will ensure that psychological distress is monitored each week, and a measure of distress conducted pre- and post-intervention will be used to supplement this. School counsellors will be well trained to monitor for sudden changes in mood and potential suicidality, including training in how to respond to suicidality with the

For peer review only - http://bmjopen.bmj.com/site/about/guidelines.xhtml

individual. All staff will be trained in following appropriate referral procedures, via the supervisor and trial coordinator, should a concern for suicide or child protection concern arise.

Adverse events reporting

A critical incident register will be maintained during the study to record serious adverse events and other adverse events. Recognition and management of all serious adverse events and other adverse events will be included in the training of school counsellors and the research staff. This will include a series of steps which have to be followed once such an event occurs including their detection, classification, reporting requirements and mitigation. Adverse events will be reported to trial ethics committees.

Data management

All quantitative research data including baseline and follow-up outcome assessment from the research participants will be collected by trained assessors electronically using tablet computers on an android based application named Open Data Kit (ODK). All research data will be remotely uploaded as a Comma Separated Values (CSV) file on the main data server running online using ODK by Assessment Team (AT) on each day of assessment. The data server will be GCP compliant including a date- and time-stamp for original data entry; an audit trail documenting any subsequent changes will be maintained. Data will be exported from the web server at the end of each day and will be checked for consistency and quality by the data manager. Exported data sets will be stored in password protected computer, only accessible by the data manager. Database will be backed-up on a daily basis and the back-up data will be stored on secure hard drives that will be stored in a secure location. Process monitoring data such as supervision attendance, number of supervision meetings conducted and data of number of intervention strategies implemented will be collected in paper form, and this will be manually entered and stored as CSV files. All quantitative research data will be deidentified.

The qualitative data will be collected in audio and paper formats and will be stored in locked filling cabinets. It will be manually entered for analysis by separating the identifying information such as participants' names and school name from the main data. Identifiable data will never be stored on portable devices unless those devices are stored in secure physical storage. Appropriate firewalls, encryption, and password protection will be used for network-connected devices used to store study data. Qualitative data will be recorded on digital recorders with the informed consent of participants, and at the end of each day audio files will be transferred to computers and deleted from portable devices, and stored in a password protected folders on computers that are backed-up daily. The audio files will be destroyed after transcription.

Statistical analysis

The findings of the trial will be reported following the updated recommendations of the CONSORT 2010 statement: extension to cluster randomized trials (36). This will include the flow of clusters and research participants through each stage of the trial, including the number eligible, randomly assigned, receiving the intended treatment, completing the study protocol and analysed for the primary outcome. Initial analyses will compare baseline characteristics of research participants across the two study arms; participants who complete follow-up assessments and participants who could not complete follow up assessments; and a comparison of the distribution of potential confounding factors. The outcome measures will be summarized at baseline and 3-month post-intervention follow-up by intervention arm and overall. These will be summarized by means (SD), medians (IQR) or numbers and proportions as appropriate (and including age, gender,

For peer review only - http://bmjopen.bmj.com/site/about/guidelines.xhtml

baseline outcome score), adjusting for cluster. Data will be cleaned and checked for accuracy prior to analysis. The consort flow for the trial is given below (Figure 1).

For the analysis of the primary outcome (reduction in PSC psychosocial distress scores from baseline to 3-months' post intervention follow-up), a linear mixed model will be employed with treatment, visit, interaction between treatment and visit as fixed effects, gender and the baseline value of the PSC psychosocial distress score as covariates, subject and cluster (school cluster) as random effects. In addition, adjusted linear mixed model analysis will be performed with the pre-specified covariates (parent/primary caregivers' education) measured at baseline being added into the above linear mixed model, which will be identified and listed in the statistical analysis plan. The crude and adjusted mean differences in the primary outcome together with its 95% confidence intervals between intervention and control at 3-months will be derived from the mixed models. In addition, subgroup analysis of primary endpoint will be performed on the above pre-specified covariates. Analysis of secondary continuous outcomes with single follow-up measurement will be done using a linear mixed model with treatment as fixed effect, gender and the baseline value of the outcome variable as covariates, and cluster (school cluster) as random effect. Analysis of secondary continuous outcomes with repeated follow-up measurement will be performed in a similar fashion as the primary endpoint analysis. The analysis of binary outcomes will use a generalized linear mixed model with treatment as fixed effect, baseline measurement of the outcome variable and gender as covariates, subject and cluster (school cluster) as random effects. The generalized linear mixed model will have a binomial distribution and logit link function, which will generate odds ratios with their 95% confidence intervals of having an event between intervention and control.

Primary data analyses will be based on the intention-to-treat principle. The per-protocol analyses will also be performed as supplemental analysis. Descriptive statistics will be produced for outcome variables and also for baseline characteristics of participants by treatment arm and visit. Continuous variables will be summarized using number of observations, mean, median, standard deviation, min and max by treatment arm and visit; categorical variables will be summarized by the number and percentage of research participants with mental health problems by treatment arm and visit. Adjusted analysis and subgroup analysis will be based on covariates at baseline without non-missing values (37). Detailed imputation methods will be described in the statistical analysis plan. All analyses will be detailed in the statistical analysis plan which will be finalized before the un-blinding of the study. No interim analysis of outcomes is planned.

We will conduct a cost-effectiveness analysis to evaluate the cost-effectiveness of EASE intervention in improving outcomes. We will calculate service use for each participant using the data from Client Services Receipt Inventory (CSRI) (34). Service utilisation and the out-of-pocket expenditures of the participants (costs for seeing a doctor or other health-care provider, admission to hospital, medicines, tests and extra help at home) will be collected at baseline and 3-months post-intervention. The data collected through the CSRI will be used to calculate service costs and total costs of care for each participant. Unit costs of services itemised in the CSRI – such as cost per outpatient visit – will be based on locally conducted health facility costing exercises.

Service cost data will subsequently be linked to primary and secondary study outcomes, in particular internalizing symptoms scores to assess issues around the value or cost-effectiveness of the EASE intervention. In the event that dominance is not shown, i.e., the EASE intervention is more effective but the costs are also more than in the wait-list group, incremental cost-effectiveness ratios will be computed, together with their confidence intervals (using bootstrapping techniques

For peer review only - http://bmjopen.bmj.com/site/about/guidelines.xhtml

to overcome the expected skewness of the cost data). Results will be plotted on a cost-effectiveness plane and presented as cost-effectiveness acceptability curves to show the probability of the intervention being cost-effective at a range of willingness-to-pay threshold levels. A sensitivity analysis will be conducted to take account of uncertainty and imprecision in the measurements, including multiple imputation models for missing values.

Qualitative process evaluation

Qualitative methods will be used to assess assumptions underlying the intervention strategy. In-Depth Interviews (IDIs) and Focus Group Discussion (FGDs) will explore key program implementation outcome variables and will cover intervention: acceptability, feasibility, appropriateness (including cultural appropriateness), fidelity, adoption and participants' view about intervention's perceived impact (both negative and positive) and ethics and safety concerns (Proctor, 2009). Following well-established procedures, qualitative interviews will be recorded, transcribed in Urdu and analysed in the original language (translation into English will only take place for the purpose of international reporting). IDIs and FGDs will be conducted at the preferred venue of the respondents, whether at home, at school, or at any other place of convenience where privacy for IDIs / FDGs can be assured.

Sampling: Interviews will be conducted with both adolescents and caregivers in the intervention including completers and drop outs, non-specialist facilitators, supervisory and school staff (teachers and head teachers). Sampling for qualitative interviews will be purposive based upon the knowledge and exposure to EASE for each category of respondent. Sampling for in-depth interviews will continue until theoretical saturation has been reached, anticipated to require 8 – 15 interviews with each category of respondent.

IDIs and FGDs will be conducted by the qualitative research team who will be trained in the key principles of qualitative interviewing. One interviewer will ask the questions and the other will take notes of the interview. Audio recordings will also be taken. All data will be anonymised and no identifying information will be collected during the interview. The interviews will follow a semi-structured interview guide addressing topics relevant to each category of respondents (Table 2).

Table 2: EASE semi structured interview summary guide

Sample	Themes
Non-specialist facilitators (delivery agents in EASE)	Intervention's acceptability, feasibility, appropriateness (including cultural appropriateness), fidelity, adoption, intervention's perceived impact (both negative and positive), ethics and safety concerns
Beneficiaries (adolescents in EASE)	Intervention's acceptability, feasibility, appropriateness (including cultural appropriateness), adoption, intervention's perceived impact (both negative and positive) and safety concerns
Beneficiaries (caregivers in EASE)	Intervention's acceptability, feasibility, appropriateness (including cultural appropriateness), adoption, intervention's perceived impact (both negative and positive) and safety concerns
Supervisory staff	Intervention's acceptability, feasibility, appropriateness (including cultural appropriateness), fidelity, adoption, intervention's perceived impact (both negative and positive), ethics and safety concerns
School staff	Barriers and facilitator of implementing intervention in school settings including perceived impact of intervention (both negative and positive)

For peer review only - http://bmjopen.bmj.com/site/about/guidelines.xhtml

Analysis of in-depth interviews will be thematic, aided by application of the framework approach (38), which complements applied research, offers transparency in the analysis process, and has an ability to move from descriptive narrative accounts to conceptual explanations. Analysis will move through phases of familiarisation, generation of codes, and selection of illustrative quotes (39), conducted by multiple members of the qualitative research team. Data from FDGs and IDIs will be triangulated to ensure a comprehensive understanding through convergence or divergence of findings relating to each topic explored in interviews.

Trial management

The Trial Steering Committee (TSC) comprising of Principal Investigator (PI), Co-Investigator (s), trial coordinator, senior researchers and intervention staff who will meet monthly to oversight the study and manage the trial.

Ethics

Ethical approval for the current study was obtained from Central Ethics Committee of University of Liverpool, UK, Ethics Review Committee of World Health Organization (WHO) Geneva and from Human Development Research Foundation Institutional Review Board, Islamabad, Pakistan. Data collection will be proceeded after seeking informed from the primary caregiver and assent from adolescents. All of the team members will be trained to ensure safety and confidentiality of participants throughout the research. An independent Trial Steering Committee (TSC) will be set-up to ensure human subject protection to the highest standards.

Dissemination

The dissemination of intervention will be carried through peer reviewed publications and training to the relevant institutions to inform the Education and Health Ministries of Pakistan, in order to scale-up program to public sector education. In addition to that the results will be disseminated through WHO's media channels (40). The results of this study will be disseminated in Urdu to community key stakeholders (such as participant school head teachers, children and parents/primary caregivers) through reports or community presentations.

Figure 1: Flow of participants through cRCT

Acknowledgment

The study sponsor and funders have no role in the study design; collection, management, analysis, and interpretation of data; writing of the report; and the decision to submit the report for publication.

Authors' contributions: AR (Principal Investigator) conceived the study and was involved in developing the research design and supervising all aspects of the study. SUH (Co-Principal Investigator) was involved in conception of the study, developing the research design, study protocol, finalising the study tools, training and day-to-day supervision of the study team in Pakistan and drafted the initial manuscript. DW (Co-Investigator) was responsible for all the statistical aspects of the study including development of the statistical analysis plan. ZeH (trial coordinator) worked with UH to develop the research design, study protocol, finalising the study tools, training and day-to-day supervision of the study team and drafted the manuscript. AM, ATN, UB, NS, HJ, MvO, SM, SAK and FAM contributed to the writing.
All authors read and approved the final manuscript.

For peer review only - http://bmjopen.bmj.com/site/about/guidelines.xhtml

Funding: The project is co-funded by the Medical Research Council (MRC) and the Department of Health Social Care (DHSC) through its National Institutes of Health Research (NIHR) via grant award Reference MR/R022410/1.

Competing interests: None declared.

References

1. Fazel M, Hoagwood K, Stephan S, Ford T. Mental health interventions in schools 1: Mental health interventions in schools in high-income countries. The lancet Psychiatry. 2014;1(5):377-87.
2. Fazel M, Patel V, Thomas S, Tol W. Mental health interventions in schools in low-income and middle-income countries. The lancet Psychiatry. 2014;1(5):388-98.
3. Barry MM, Clarke AM, Jenkins R, Patel V. A systematic review of the effectiveness of mental health promotion interventions for young people in low and middle income countries. BMC public health. 2013;13:835.
4. Kumar D. School mental health program in India: Need to shift from a piecemeal approach to a long-term comprehensive approach with strong intersectoral coordination. Indian J Psychiatry. 2021;63(1):91-6.
5. Lee J. Mental health effects of school closures during COVID-19. The Lancet Child & adolescent health. 2020;4(6):421.
6. Jones EAK, Mitra AK, Bhuiyan AR. Impact of COVID-19 on Mental Health in Adolescents: A Systematic Review. Int J Environ Res Public Health. 2021;18(5):2470.
7. Hamdani SU, Huma ZE, Tamizuddin-Nizami A. Debate: Child and adolescent mental health services in Pakistan; Do we need in-patient mental health facilities for children and young people? Child and adolescent mental health. 2021;26(2):182-3.
8. Mian AI, Chachar AS. Debate: COVID-19 and school mental health in Pakistan. Child and adolescent mental health. 2020;25(4):270-2.
9. Hamdani SU, Huma ZE, Javed H, Warraitch A, Rahman A, Tameezuddin Nizami A, et al. Prevalence of psychosocial distress in school going adolescents in rural Pakistan: findings from a cross-sectional epidemiological survey. BJPsych Open. 2021;7(S1):S56-S7.
10. Mirza Z, Rahman A. Mental health care in Pakistan boosted by the highest office. Lancet (London, England). 2019;394(10216):2239-40.
11. Dawson KS, Watts S, Carswell K, Shehadeh MH, Jordans MJ, Bryant RA, et al. Improving access to evidence-based interventions for young adolescents: Early Adolescent Skills for Emotions (EASE). World Psychiatry. 2019;18(1):105.
12. Hamdani SU, Huma ZE, Suleman N, Warraitch A, Muzzafar N, Farzeen M, et al. Scaling-up school mental health services in low resource public schools of rural Pakistan: the Theory of Change (ToC) approach. International journal of mental health systems. 2021;15(1):8.
13. Population Council [Pakistan]. Landscape Analysis of Family Planning Situation in Pakistan: Brief Summary of Findings: District Population Council; 2016.
14. Durlak JA, Weissberg RP, Dymnicki AB, Taylor RD, Schellinger KB. The impact of enhancing students' social and emotional learning: A meta-analysis of school-based universal interventions. Child development. 2011;82(1):405-32.
15. Buchanan-Pascall S, Gray KM, Gordon M, Melvin GA. Systematic review and meta-analysis of parent group interventions for primary school children aged 4–12 years with

For peer review only - http://bmjopen.bmj.com/site/about/guidelines.xhtml

externalizing and/or internalizing problems. Child Psychiatry & Human Development. 2018;49(2):244-67.
16. Organization WH. World health statistics 2016: monitoring health for the SDGs sustainable development goals: World Health Organization; 2016.
17. Kohrt BA, Jordans MJ, Rai S, Shrestha P, Luitel NP, Ramaiya MK, et al. Therapist competence in global mental health: development of the ENhancing Assessment of Common Therapeutic factors (ENACT) rating scale. Behaviour research and therapy. 2015;69:11-21.
18. Jellinek MS, Murphy JM, Robinson J, Feins A, Lamb S, Fenton T. Pediatric Symptom Checklist: screening school-age children for psychosocial dysfunction. The Journal of pediatrics. 1988;112(2):201-9.
19. Y-PSC P. Pediatric Symptom Checklist. Journal of Developmental and Behavioral Pediatrics. 1994;15:191-7.
20. Trickey D. RCADS: The Revised Children's Anxiety and Depression Scale(RCADS) and-The Revised Children's Anxiety and Depression Scale–Parent Version (RCADS-P). Guide to Using Outcomes and Feedback Tools. 2014:116.
21. D'Zurilla TJ, Nezu AM, Maydeu-Olivares A. Social problem-solving inventory-revised. 2002.
22. Chaudhry N, Rasool G. A case study on improving problem solving skills of undergraduate computer science students. World Applied Sciences Journal. 2012;20(1):34-9.
23. Slavin LA. Validation Studies of the PEPSS, a Measure of Perceived Emotional Support for Use with Adolescents. Journal of Adolescent Research. 1991;6(3):316-35.
24. Richardson LP, McCauley E, Grossman DC, McCarty CA, Richards J, Russo JE, et al. Evaluation of the Patient Health Questionnaire-9 Item for detecting major depression among adolescents. Journal of Pediatrics. 2010;126(6):1117-23.
25. Tennant R, Hiller L, Fishwick R, Platt S, Joseph S, Weich S, et al. The Warwick-Edinburgh mental well-being scale (WEMWBS): development and UK validation. Journal of Health and Quality of Life Outcomes. 2007;5(1):63.
26. Vaingankar JA, Abdin E, Chong SA, Sambasivam R, Seow E, Jeyagurunathan A, et al. Psychometric properties of the short Warwick Edinburgh mental well-being scale (SWEMWBS) in service users with schizophrenia, depression and anxiety spectrum disorders. Health and quality of life outcomes. 2017;15(1):1-11.
27. Varni JW, Seid M, Rode CA. The PedsQL™: measurement model for the pediatric quality of life inventory. Medical care. 1999:126-39.
28. Moses T. Self-labeling and its effects among adolescents diagnosed with mental disorders. Social science & medicine. 2009;68(3):570-8.
29. Khalil A, Gondal F, Imran N, Azeem MW. Self-Stigmatization in children receiving mental health treatment in Lahore, Pakistan. Asian journal of psychiatry. 2020;47:101839.
30. Varni JW, Seid M, Rode CA. The PedsQL™: measurement model for the pediatric quality of life inventory. Journal of Medical Care. 1999:126-39.
31. Zlomke KR, Lamport D, Bauman S, Garland B, Talbot B. Parenting Adolescents: Examining the Factor Structure of the Alabama Parenting Questionnaire for Adolescents. Journal of Child and Family Studies. 2014;23(8):1484-90.
32. Elgar FJ, Waschbusch DA, Dadds MR, Sigvaldason N. Development and validation of a short form of the Alabama Parenting Questionnaire. Journal of Child and Family Studies. 2007;16(2):243-59.

For peer review only - http://bmjopen.bmj.com/site/about/guidelines.xhtml

33. Brown FL, Steen F, Taha K, Aoun M, Bryant RA, Jordans MJD, et al. Early Adolescent Skills for Emotions (EASE) intervention for the treatment of psychological distress in adolescents: study protocol for randomised controlled trials in Lebanon and Jordan. Trials. 2019;20(1):545.
34. Chisholm D, Knapp MRJ, Knudsen HC, Amaddeo F, Gaite L, Van Wijngaarden B, et al. Client socio-demographic and service receipt inventory–European version: development of an instrument for international research: EPSILON Study 5. The British Journal of Psychiatry. 2000;177(S39):s28-s33.
35. Godfrey E, Aubrey M, Crockford S, Haythorne D, Kordowicz M, Ashworth M. The development and testing of PSYCHLOPS Kids: a new child-centred outcome measure. Child and adolescent mental health. 2019;24(1):54-65.
36. Campbell MK, Piaggio G, Elbourne DR, Altman DG. Consort 2010 statement: extension to cluster randomised trials. Bmj. 2012;345.
37. Enders C. Applied missing data analysis. 2010. New York: GuilfordGoogle Scholar.
38. Furber C. Framework analysis: a method for analysing qualitative data. African Journal of Midwifery and Women's health. 2010;4(2):97-100.
39. Green J, Thorogood N. Qualitative methods for health research: sage; 2018.
40. WHO. mhGAP intervention guide for mental, neurological and substance use disorders in non-specialized health settings: mental health Gap Action Programme (mhGAP): World Health Organization; 2016.

For peer review only - http://bmjopen.bmj.com/site/about/guidelines.xhtml

Figure 1: Flow of participants through cRCT

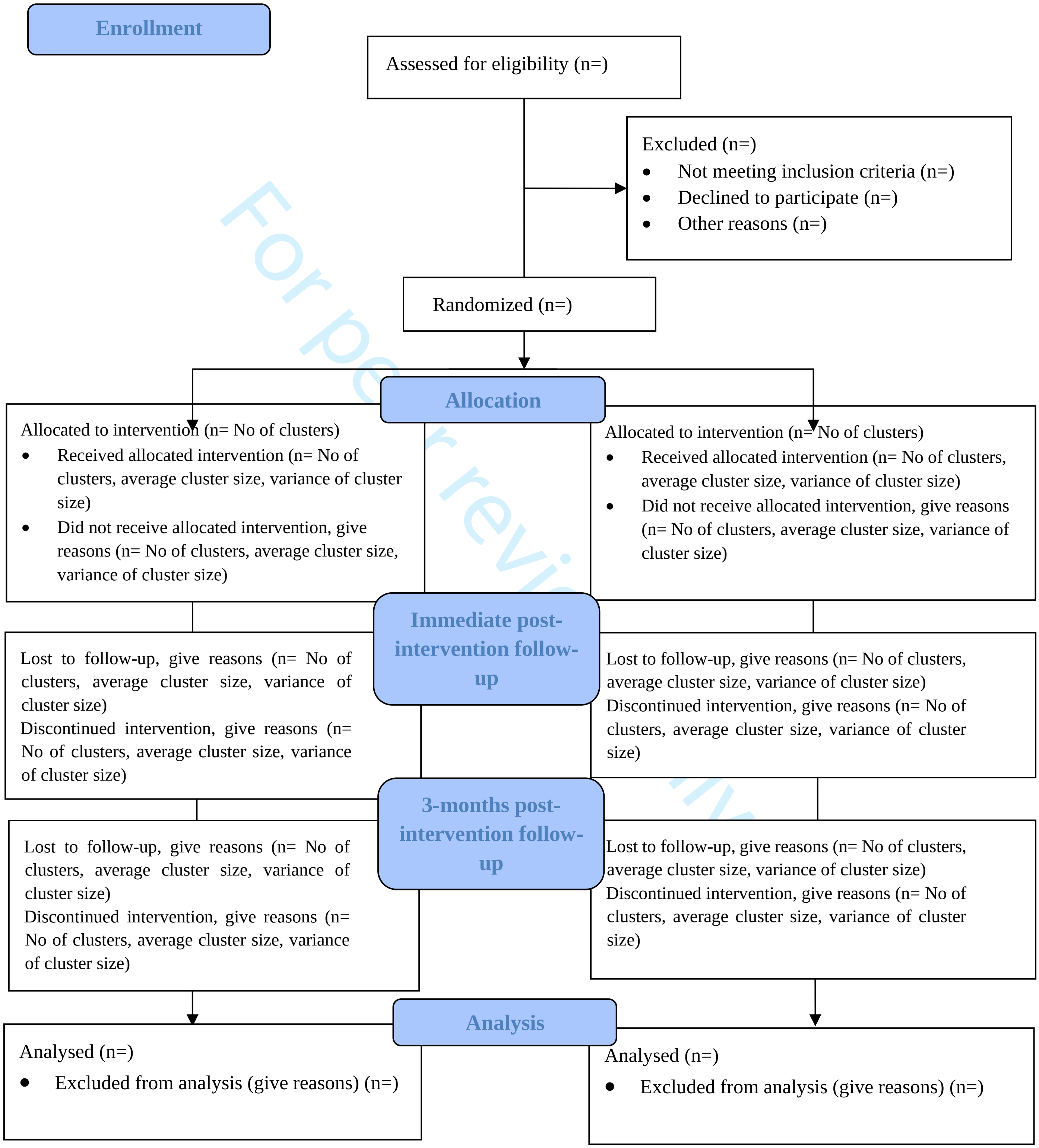

For peer review only - http://bmjopen.bmj.com/site/about/guidelines.xhtml

STANDARD PROTOCOL ITEMS: RECOMMENDATIONS FOR INTERVENTIONAL TRIALS

SPIRIT 2013 Checklist: Recommended items to address in a clinical trial protocol and related documents*

Section/item	Item No	Description	Addressed on page number
**Administrative information**			
Title	1	Descriptive title identifying the study design, population, interventions, and, if applicable, trial acronym	Title p.1
Trial registration	2a	Trial identifier and registry name. If not yet registered, name of intended registry	Abstract p. 2
	2b	All items from the World Health Organization Trial Registration Data Set	Included in the registration
Protocol version	3	Date and version identifier	N/A
Funding	4	Sources and types of financial, material, and other support	19
Roles and responsibilities	5a	Names, affiliations, and roles of protocol contributors	1 & 18
	5b	Name and contact information for the trial sponsor	N/A
	5c	Role of study sponsor and funders, if any, in study design; collection, management, analysis, and interpretation of data; writing of the report; and the decision to submit the report for publication, including whether they will have ultimate authority over any of these activities	18
	5d	Composition, roles, and responsibilities of the coordinating centre, steering committee, endpoint adjudication committee, data management team, and other individuals or groups overseeing the trial, if applicable (see Item 21a for data monitoring committee)	15

For peer review only - http://bmjopen.bmj.com/site/about/guidelines.xhtml

**Introduction**			
Background and rationale	6a	Description of research question and justification for undertaking the trial, including summary of relevant studies (published and unpublished) examining benefits and harms for each intervention	6-7
	6b	Explanation for choice of comparator	10
Objectives	7	Specific objectives or hypotheses	7
Trial design	8	Description of trial design including type of trial (eg, parallel group, crossover, factorial, single group), allocation ratio, and framework (eg, superiority, equivalence, noninferiority, exploratory)	7 & 14
**Methods: Participants, interventions, and outcomes**			
Study setting	9	Description of study settings (e.g., community clinic, academic hospital) and list of countries where data will be collected. Reference to where list of study sites can be obtained	7&8
Eligibility criteria	10	Inclusion and exclusion criteria for participants. If applicable, eligibility criteria for study centres and individuals who will perform the interventions (eg, surgeons, psychotherapists)	8
Interventions	11a	Interventions for each group with sufficient detail to allow replication, including how and when they will be administered	9-10
	11b	Criteria for discontinuing or modifying allocated interventions for a given trial participant (eg, drug dose change in response to harms, participant request, or improving/worsening disease)	N/A
	11c	Strategies to improve adherence to intervention protocols, and any procedures for monitoring adherence (eg, drug tablet return, laboratory tests)	N/A
	11d	Relevant concomitant care and interventions that are permitted or prohibited during the trial	10
Outcomes	12	Primary, secondary, and other outcomes, including the specific measurement variable (eg, systolic blood pressure), analysis metric (eg, change from baseline, final value, time to event), method of aggregation (eg, median, proportion), and time point for each outcome. Explanation of the clinical relevance of chosen efficacy and harm outcomes is strongly recommended	10-13
Participant timeline	13	Time schedule of enrolment, interventions (including any run-ins and washouts), assessments, and visits for participants. A schematic diagram is highly recommended (see Figure)	11

For peer review only - http://bmjopen.bmj.com/site/about/guidelines.xhtml

Sample size	14	Estimated number of participants needed to achieve study objectives and how it was determined, including clinical and statistical assumptions supporting any sample size calculations	8-9
Recruitment	15	Strategies for achieving adequate participant enrolment to reach target sample size	9
**Methods: Assignment of interventions (for controlled trials)**			
Allocation:			
Sequence generation	16a	Method of generating the allocation sequence (e.g., computer-generated random numbers), and list of any factors for stratification. To reduce predictability of a random sequence, details of any planned restriction (eg, blocking) should be provided in a separate document that is unavailable to those who enrol participants or assign interventions	14
Allocation concealment mechanism	16b	Mechanism of implementing the allocation sequence (e.g., central telephone; sequentially numbered, opaque, sealed envelopes), describing any steps to conceal the sequence until interventions are assigned	14
Implementation	16c	Who will generate the allocation sequence, who will enrol participants, and who will assign participants to interventions	14
Blinding (masking)	17a	Who will be blinded after assignment to interventions (eg, trial participants, care providers, outcome assessors, data analysts), and how	14
	17b	If blinded, circumstances under which unblinding is permissible, and procedure for revealing a participant's allocated intervention during the trial	14
**Methods: Data collection, management, and analysis**			
Data collection methods	18a	Plans for assessment and collection of outcome, baseline, and other trial data, including any related processes to promote data quality (eg, duplicate measurements, training of assessors) and a description of study instruments (eg, questionnaires, laboratory tests) along with their reliability and validity, if known. Reference to where data collection forms can be found, if not in the protocol	15&16
	18b	Plans to promote participant retention and complete follow-up, including list of any outcome data to be collected for participants who discontinue or deviate from intervention protocols	N/A

For peer review only - http://bmjopen.bmj.com/site/about/guidelines.xhtml

Data management	19	Plans for data entry, coding, security, and storage, including any related processes to promote data quality (eg, double data entry; range checks for data values). Reference to where details of data management procedures can be found, if not in the protocol	15&16
Statistical methods	20a	Statistical methods for analysing primary and secondary outcomes. Reference to where other details of the statistical analysis plan can be found, if not in the protocol	15-17
	20b	Methods for any additional analyses (eg, subgroup and adjusted analyses)	15-17
	20c	Definition of analysis population relating to protocol non-adherence (eg, as randomised analysis), and any statistical methods to handle missing data (eg, multiple imputation)	16-17
**Methods: Monitoring**			
Data monitoring	21a	Composition of data monitoring committee (DMC); summary of its role and reporting structure; statement of whether it is independent from the sponsor and competing interests; and reference to where further details about its charter can be found, if not in the protocol. Alternatively, an explanation of why a DMC is not needed	18-19
	21b	Description of any interim analyses and stopping guidelines, including who will have access to these interim results and make the final decision to terminate the trial	N/A
Harms	22	Plans for collecting, assessing, reporting, and managing solicited and spontaneously reported adverse events and other unintended effects of trial interventions or trial conduct	15
Auditing	23	Frequency and procedures for auditing trial conduct, if any, and whether the process will be independent from investigators and the sponsor	N/A
**Ethics and dissemination**			
Research ethics approval	24	Plans for seeking research ethics committee/institutional review board (REC/IRB) approval	18
Protocol amendments	25	Plans for communicating important protocol modifications (eg, changes to eligibility criteria, outcomes, analyses) to relevant parties (eg, investigators, REC/IRBs, trial participants, trial registries, journals, regulators)	N/A

For peer review only - http://bmjopen.bmj.com/site/about/guidelines.xhtml

Consent or assent	26a	Who will obtain informed consent or assent from potential trial participants or authorised surrogates, and how (see Item 32)	9
	26b	Additional consent provisions for collection and use of participant data and biological specimens in ancillary studies, if applicable	N/A
Confidentiality	27	How personal information about potential and enrolled participants will be collected, shared, and maintained in order to protect confidentiality before, during, and after the trial	15
Declaration of interests	28	Financial and other competing interests for principal investigators for the overall trial and each study site	19
Access to data	29	Statement of who will have access to the final trial dataset, and disclosure of contractual agreements that limit such access for investigators	18
Ancillary and post-trial care	30	Provisions, if any, for ancillary and post-trial care, and for compensation to those who suffer harm from trial participation	N/A
Dissemination policy	31a	Plans for investigators and sponsor to communicate trial results to participants, healthcare professionals, the public, and other relevant groups (eg, via publication, reporting in results databases, or other data sharing arrangements), including any publication restrictions	18
	31b	Authorship eligibility guidelines and any intended use of professional writers	N/A
	31c	Plans, if any, for granting public access to the full protocol, participant-level dataset, and statistical code	N/A
**Appendices**			
Informed consent materials	32	Model consent form and other related documentation given to participants and authorised surrogates	N/A
Biological specimens	33	Plans for collection, laboratory evaluation, and storage of biological specimens for genetic or molecular analysis in the current trial and for future use in ancillary studies, if applicable	N/A

*It is strongly recommended that this checklist be read in conjunction with the SPIRIT 2013 Explanation & Elaboration for important clarification on the items. Amendments to the protocol should be tracked and dated. The SPIRIT checklist is copyrighted by the SPIRIT Group under the Creative Commons "Attribution-NonCommercial-NoDerivs 3.0 Unported" license.

For peer review only - http://bmjopen.bmj.com/site/about/guidelines.xhtml
