# Supplementary material for: Improving psychosocial distress for young adolescents in rural schools of Pakistan: study protocol of a cluster randomised controlled trial
Source: BMJ Open. 2022 Sep 23;12(9):e063607. doi: 10.1136/bmjopen-2022-063607 (PMC9511576; doi:10.1136/bmjopen-2022-063607)
Supplement: Reviewer comments [file bmjopen-2022-063607.reviewer_comments.pdf]

PEER REVIEW HISTORY

BMJ Open publishes all reviews undertaken for accepted manuscripts. Reviewers are asked to complete a checklist review form (**http://bmjopen.bmj.com/site/about/resources/checklist.pdf**) and are provided with free text boxes to elaborate on their assessment. These free text comments are reproduced below.

ARTICLE DETAILS

**TITLE (PROVISIONAL)**	Improving psychosocial distress for young adolescents in rural schools of Pakistan: Study protocol of a cluster Randomized Controlled Trial
**AUTHORS**	Hamdani, Syed Usman; Huma, Zill-e-; Malik, Aiysha; Nizami, Asad; Baneen, Um ul; Suleman, Nadia; Javed, Hashim; Wang, Duolao; Van Ommeren, Mark; Mazhar, Samra; Khan, Shahzad; Minhas, Fareed; Rahman, Atif

This article was not externally reviewed at BMJ Open. Protocols that have been independently assessed prior to submission to BMJ Open are usually fast-tracked to publication on the grounds that further substantial changes will not be possible. This independent assessment will usually be external, independent review for both a substantial grant award from a non-commercial or government funder and ethics approval.
